# Fourteen year retrospective study of craniofacial pain in a neurological emergency department

**DOI:** 10.1038/s41598-025-01246-1

**Published:** 2025-09-30

**Authors:** Aleksandra Lučić, Zlatko Božić, Aleksandar Kopitović, Sanela Popović, Dane Krtinić, Igor Petrušić

**Affiliations:** 1 Faculty of Medicine, University of Novi Sad, Hajduk Veljkova 3, Novi Sad, 21000 Serbia; 2 Neurology clinic, University Clinical Center of Vojvodina, Hajduk Veljkova 1, Novi Sad, 21137 Serbia; 3 Department for pharmacology and toxicology, Faculty of Medicine, University of Niš, Blvd. Dr Zorana Đinđića 81, Niš, 18108 Serbia; 4 Clinic for Oncology, University Clinical Center Niš, Blvd. Dr Zorana Đinđića 48, Niš, 18108 Serbia; 5 Laboratory for Advanced Analysis of Neuroimages, Faculty of Physical Chemistry, University of Belgrade, Studentski trg 12-16, 11158 Belgrade 118, Belgrade, Serbia

**Keywords:** Facial pain, Trigeminal neuralgia, Cross-Sectional studies, Pain management, Emergency service, hospital, Neuropathic pain, Headache, Pain, Chronic pain

## Abstract

This fourteen-year retrospective cross-sectional study (August 2010 - July 2024) provides the first analysis of neuropathic craniofacial pain (CFP) in a neurological emergency department (ED), evaluating its types, characteristics, and management. Data from electronic medical records were analyzed using descriptive statistics and non-parametric tests (*p* < 0.05). Among 156 adult CFP patients (median age 61 years; female 108/156, 69.2%), six CFP types were identified, with trigeminal neuralgia (TN) being the most common (122/156, 78.2%), and etiologically defined in 30/122 patients (24.6%). The typical patient was an elderly female with severe, right-sided TN affecting the V2 and V3 branches. Acute treatment lacked guideline-supported rapid-onset options, relying instead on nonopioid and opioid analgesics (48/54 patients, 88.9%). There was a decline in cases after the beginning of the COVID-19 pandemic (*p* = 0.001). Rare CFPs and their combinations can initially present in the ED, where diagnostic complexity and treatment gaps pose significant challenges. These findings highlight the ED’s critical role and the need for institutional guidelines based on ICHD-3 or ICOP to improve CFP management.

**Supplementary Information:**

The online version contains supplementary material available at 10.1038/s41598-025-01246-1.

## Introduction

Pain, including craniofacial pain (CFP), is among the most common reasons for emergency department (ED) visits^1–4^. CFP is a complex multifaceted disorder mediated by afferent fibers of the cranial nerves and upper cervical roots^4,5^. Neuralgias are the most frequent type of neuropathic CFP caused by dysfunction or injury of these neural structures^4–6^. Due to the complex neuroanatomy and specific sensory innervation of the head and neck, CFP conditions deserve special attention. Despite precise definitions in the International Classification of Headache Disorders 3rd edition (ICHD-3)^6^ and the subsequent International Classification of Orofacial Pain (ICOP)^7^, many of these disorders still pose a clinical diagnostic challenge. CFPs carry a significant patient illness burden, exacerbated by frequent diagnostic and management challenges, especially in the ED^1–3^.

Our study aimed to assess the frequency and characteristics of specific types of CFP and evaluate the treatment strategies employed in the neurological ED, alongside the challenges neurologists face in managing these cases.

## Methods

The study encompassed fourteen years, from August 2010 (when the Emergency center was opened) through July 2024, and included referred and self-referred patients with CFP who presented to the neurological ED at the Emergency Center of the UCCV. The exclusion criteria were primary and secondary headaches, other forms of non-neurological orofacial pain (isolated or concurrent), patients younger than 18 years, and reports with insufficient data to provide information about the patient, confirm the diagnosis, and rule out differential diagnosis.

All research was performed in accordance with relevant guidelines and regulations. The study was conducted in accordance with the Declaration of Helsinki and approved by the Institution’s Ethics Committee at the University Clinical Center of Vojvodina (UCCV) in Novi Sad, Serbia (00–58/2022). Due to the retrospective nature of the study, the Ethics committee of the University Clinical Center of Vojvodina waived the need to obtain informed consent.

Examination reports were reviewed for demographic data, data regarding the characteristics of pain syndrome, neuroimaging results (conducted during the ED visit or provided by patients), and surgical/invasive or pharmacological treatment. To minimize observer bias, all patient records were initially reviewed independently by Z.B. and S.P. In cases where the diagnosis or classification was unclear, a secondary review was conducted by A.L. and A.K. Discrepancies between the two reviewers were resolved through discussion with other authors until a consensus was reached. This process ensured consistency and reliability in data classification. After review, patients were classified according to their discharge diagnosis. Considering certain diagnostic difficulties in the ED, the authors added the prefix “presumed” to some diagnoses. In the case of presumed occipital neuralgia (pON), it was due to the absence of a confirmatory test (criterion D in ICHD-3), and in the case of presumed persistent idiopathic facial pain (pPIFP) it was due to the unmet pain duration criterion and incompletely excluded alternative diagnoses (criteria B and E according to ICHD3). For presumed trigeminal and glossopharyngeal neuralgia (pTN + pGN), criteria B and C were not described in the report. There was no patient or public involvement in the research process.

The variable ‘sex’ in this study was determined based on information from medical records, including names and legal identifiers. We did not have access to patients’ self-reported sex or gender identity. As such, the categorization reflects the assigned sex as recorded in legal documents, which may not correspond to patients’ self-identified gender.

Patients were divided into four groups based on the disease duration: less than a week, one week but less than three months, three months and up to one year, and more than a year. The course was defined based on Maarbjerg et al.^8^ as a monophasic-acute, relapsing-remitting, and chronic course. The pain intensity groups were defined as mild (1–3), moderate (4–6), and severe pain (7–10).

Patients were divided into two groups (pre and post) based on the disease onset relative to the first registered COVID-19 case in Serbia (6th March 2020). This was used for analysis of differences in CFP characteristics. Additionally, patients were grouped according to whether they reported to ED before or after the onset of the COVID-19 pandemic (pre-COVID-19: August 2010 - March 2020, post-COVID-19: March 2020 - July 2024). This second grouping was used to assess whether COVID-19 had an impact on the frequency of ED visits. The test compared the observed frequency of patients in the pre-COVID-19 period to the expected proportion of 0.69, adjusted for the difference in period duration.

After extraction, the data were coded and entered into the database. Statistical processing was performed using the software Statistical Package for Social Sciences (SPSS), version 23.0 (IBM Corp., Armonk, NY).

The results are presented by methods of descriptive statistics: frequencies and percentages; median and interquartile range for non-normally distributed data. Data normality was assessed by histogram visual inspection and the Shapiro-Wilk test. Fisher’s exact test was used to analyze differences in the frequency of SD in TN patients depending on previous invasive treatment. A binomial test was used to assess the difference in frequency of CFP and TN patients in relation to the first case of COVID-19 in Serbia. The statistical analysis methodology and the results regarding differences between characteristics of CFP diagnosed before and after COVID-19 onset are shown in the Supplementary Data Tables 3–9. The p-value of < 0.05 was considered statistically significant. Missing data is reported in Supplementary Data Table 1.

**Table 1:** Demographic and clinical characteristics of the study population and craniofacial pain types.

Group, n (%^a^)		**Total, 156**	**TN, 122 (78.2)**	**TNp, 11 (7.1)**	**pON, 10 (6.4)**	**pPIFP, 4 (2.6)**	**GN, 4 (2.6)**	**NINp, 4 (2.6)**	**pTN+pGN, 1 (0.6)**
Age at presentation (y) median, IQR		61, 43–73	61, 41–72	71, 58–75	64, 46–69	36, 33–42	61, 51–78	67, 59–78	86
Age at onset (y) median, IQR		56, 40–69	56, 38–68	66, 51–75	65, 46–69	36, 33–42	58, 51–75	67, 59–78	86
Sex	Female	108 (69.2)	84 (68.9)	5 (45.5)	8 (80.0)	4 (100.0)	3 (75.0)	3 (75.0)	1 (100.0)
Duration	< 1 week	26 (16.7)	16 (13.1)	2 (18.2)	4 (40.0)	2 (50.0)	0	1 (25.0)	1 (100.0)
	1 week – 3 months	36 (23.1)	20 (16.4)	5 (45.5)	3 (30.0)	2 (50.0)	3 (75.0)	3 (75.0)	0
	3 months – 1 year	18 (11.5)	17 (13.9)	1 (9.1)	0	0	0	0	0
	> 1 year	68 (43.6)	64 (52.5)	2 (18.2)	1 (10.0)	0	1 (25.0)	0	0
Course	Monophasic - acute	64 (41.0)	37 (30.3)	8 (72.7)	7 (70.0)	4 (100.0)	3 (75.0)	4 (100.0)	1 (100.0)
	Relapsing - remitting	68 (43.6)	64 (52.5)	1 (9.1)	3 (30.0)	0	0	0	0
	Chronic	24 (15.4)	21 (17.2)	2 (18.2)	0	0	1 (25.0)	0	0
Affected Side	Right	93 (59.6)	73 (59.8)	6 (54.5)	7 (70.0)	2 (50.0)	1 (25.0)	3 (75.0)	1 (100.0)
	Left	56 (35.9)	47 (38.5)	3 (27.3)	2 (20.0)	1 (25.0)	2 (50.0)	1 (25.0)	0
	Both	5 (3.2)	1 (0.8)	2 (18.2)	0	1 (25.0)	1 (25.0)	0	0
Pain Intensity	Mild	3 (1.9)	3 (2.5)	0	0	0	0	0	0
	Moderate	37 (23.7)	25 (20.5)	6 (54.5)	2 (20.0)	2 (50.0)	2 (50.0)	0 (0.0)	0
	Severe	70 (44.9)	59 (48.4)	3 (27.3)	4 (40.0)	1 (25.0)	0 (0.0)	2 (50.0)	1 (100.0)
Trigger	Present	83 (53.2)	71 (58.2)	2 (18.2)	5 (50.0)	2 (50.0)	3 (75.0)	0	0
CAS	Present	22 (14.1)	18 (14.8)	2 (18.2)	2 (20.0)	0	0	0	0
SD	Present	54 (34.6)	41 (33.6)	5 (45.5)	4 (40.0)	1 (25.0)	1 (25.0)	2 (50.0)	0
Treated during visit	No therapy	102 (65.4)	80 (65.6)	7 (63.6)	5 (50.0)	3 (75.0)	4 (100.0)	3 (75.0)	0
	Monoterapy	34 (21.8)	25 (20.5)	3 (27.3)	3 (30.0)	1 (25.0)	0	1 (25.0)	1 (100.0)
	Polytherapy	20 (12.8)	17 (13.9)	1 (9.1)	2 (20.0)	0	0	0	0
Therapy at discharge	No therapy	4 (2.6)	2 (1.6)	1 (9.1)	0	0	0	1 (25.0)	0
	Monoterapy	59 (37.8)	51 (41.8)	1 (9.1)	4 (40.0)	0	2 (50.0)	0	1 (100.0)
	Polytherapy	92 (59.0)	68 (55.7)	9 (81.8)	6 (60.0)	4 (100.0)	2 (50.0)	3 (75.0)	0

a-percentages calculated by total number of patients in a selected group; TN – trigeminal neuralgia, TNp – painful trigeminal neuropathy, pON – presumed occipital neuralgia, pPIFP – presumed persistent idiopathic facial pain, GN –glossopharyngeal neuralgia; NINp –painful nervus intermedius neuropathy; pTN+pGN – presumed concomitant trigeminal and glossopharyngeal neuralgia; IQR – interquartile range; CAS – cranial autonomic symptoms, SD – sensory disturbances; Sums of % for certain groups are not necessarily 100. Missing data is reported in the Supplementary Data Table 1.

**Table 2.** Treatment in the emergency department.

Group, n (%^a^)	**Total, 156**	**TN, 122 (78.2)**	**TNp, 11 (7.1)**	**pON, 10 (6.4)**	**pPIFP, 4 (2.6)**	**GN, 4 (2.6)**	**NINp, 4 (2.6)**	**pTN+pGN, 1 (0.6)**
Coanalgesics	8 (5.1)	7 (5.7)	1 (9.1)	0	0	0	0	0
Carbamazepine	2 (1.3)	2 (1.6)	0	0	0	0	0	0
Diazepam	4 (2.6)	3 (2.5)	1 (9.1)	0	0	0	0	0
Corticosteroids	7 (4.5)	6 (4.9)	0	1 (10.0)	0	0	0	0
Dexamethasone	3 (1.9)	3 (2.5)	0	0	0	0	0	0
Methylprednisolone	4 (2.6)	3 (2.5)	0	1 (10.0)	0	0	0	0
Analgesics	48 (30.8)	36 (29.5)	4 (36.4)	5 (50.0)	1 (25.0)	0	1 (25.0)	1 (100.0)
Tramadol	13 (8.3)	12 (9.8)	0	1 (10.0)	0	0	0	0
Nonopioids	41 (26.3)	30 (24.6)	4 (36.4)	4 (40.0)	1 (25.0)	0	1 (25.0)	1 (100.)
Paracetamol	2 (1.3)	2 (1.6)	0	0	0	0	0	0
Ketoprofen	1 (0.6)	1 (0.8)	0	0	0	0	0	0
Nimesulid	2 (1.3)	1 (0.8)	0	1 (10.0)	0	0	0	0
Diclofenac	8 (5.1)	5 (4.1)	1 (9.1)	2 (20.0)	0	0	0	0
Ketorolac	24 (15.4)	19 (15.6)	2 (18.2)	0	1 (25.0)	0	1 (25.0)	1 (100.0)
Metamizole	11 (7.1)	8 (6.6)	1 (9.1)	2 (20.0)	0	0	0	0
Other (mannitol, O2)	15 (9.6)	12 (9.8)	1 (9.1)	2 (20.0)	0	0	0	0

a-percentages calculated by total number of patients in a selected group. TN – trigeminal neuralgia, TNp – painful trigeminal neuropathy, pON – presumed occipital neuralgia, pPIFP – presumed persistent idiopathic facial pain, GN – glossopharyngeal neuralgia; NINp – painful intermedius nerve neuropathy; pTN+pGN – presumed concomitant trigeminal and glossopharyngeal neuralgia; Sums of % for certain groups are not necessarily 100. Missing data is reported in the Supplementary Data Table 1.

**Table 3:** Treatment at discharge.

Group, n (%^a^)	**Total, 156**	**TN, 122 (78.2)**	**TNp, 11 (7.1)**	**pON, 10 (6.4)**	**pPIFP, 4 (2.6)**	**GN, 4 (2.6)**	**NINp, 4 (2.6)**	**pTN+pGN, 1 (0.6)**
Coanalgesics	137 (87.8)	111 (91.0)	10 (90.9)	8 (80.0)	2 (50.0)	4 (100.0)	1 (25.0)	1 (100.0)
Anticonvulsives	132 (84.6)	108 (88.5)	9 (81.8)	8 (80.0)	2 (50.0)	4 (100.0)	0	1 (100.0)
Carbamazepine	105 (67.3)	87 (71.3)	5 (45.5)	6 (60.0)	2 (50.0)	4 (100.0)	0	1 (100.0)
Gabapentine	16 (10.3)	15 (12.3)	1 (9.1)	0	0	0	0	0
Pregabalin	22 (14.1)	15 (12.3)	4 (36.4)	3 (30.0)	0	0	0	0
Levetiracetam	1 (0.6)	1 (0.8)	0	0	0	0	0	0
Klonazepam	1 (0.6)	0	1 (9.1)	0	0	0	0	0
Valproate	1 (0.6)	1 (0.8)	0	0	0	0	0	0
Amitriptyline	13 (8.3)	10 (8.2)	2 (18.2)	0	0	0	1 (25.0)	0
Benzodiazepines	11 (7.1)	6 (4.9)	0	1 (10.0)	1 (25.0)	1 (25.0)	2 (50.0)	0
Diazepam	5 (3.2)	3 (2.5)	0	1 (10.0)	0	0	1 (25.0)0	0
Bromazepam	5 (3.2)	3 (2.5)	0	0	1 (25.0)	1 (25.0)	0	0
Lorazepam	1 (0.6)	0	0	0	0	0	1 (25.0)	0
Corticosteroids	4 (2.6)	3 (2.5)	0	0	0	0	1 (25.0)	0
Methylprednisolone	1 (0.6)	1 (0.8)	0	0	0	0	0	0
Dexamethasone	3 (1.9)	2 (1.6)	0	0	0	0	1 (25.0)	0
Analgesics	64 (41.0)	45 (36.9)	7 (63.6)	5 (50.0)	4 (100.0)	1 (25.0)	2 (50.0)	0
Opioids	21 (13.5)	18 (14.8)	1 (9.1)	2 (20.0)	0	0	0	0
Tramadol	20 (12.8)	17 (13.9)	1 (9.1)	2 (20.0)	0	0	0	0
Fentanyl	1 (0.6)	1 (0.8)	0	0	0	0	0	0
Nonopioids	56 (35.9)	38^‡^(31.1‡)	6 (54.5)	5 (50.0)	4 (100.0)	1 (25.0)	2 (50.0)	0
CAM	38 (24.4)	29 (23.8)	3 (27.3)	1 (10.0)	2 (50.0)	0	3 (75.0)	0
Capsaicine	4 (2.6)	3 (2.5)	1 (9.1)	0	0	0	0	0

a-percentages calculated by total number of patients in a selected group. TN – trigeminal neuralgia, TNp –painful trigeminal neuropathy, pON –presumed occipital neuralgia, pPIFP – presumed persistent idiopathic facial pain, GN – glossopharyngeal neuralgia; NINp – painful intermedius nerve neuropathy; pTN+pGN – presumed concomitant trigeminal and glossopharyngeal neuralgia; CAM – complementary and alternative medicine. Sums of % for certain groups are not necessarily 100. Missing data is reported in the Supplementary Data Table 1.

**Table 4:** Clinical characteristics of selected craniofacial pain categories.

Group, n (%^a^)		**TN, 122 (78.2)**	**TNp, 11 (7.1)**	**pPIFP, 4 (2.6)**
Distribution^b^	V1	10 (8.2)	7 (63.6)	0
	V2	31 (25.4)	3 (27.3)	0
	V3	20 (16.4)	0	1 (25.0)
	V1+V2	12 (9.8)	1 (9.1)	0
	V1+V3	1 (0.8)	0	0
	V2+V3	32 (26.2)	0	1 (25.0)
	V1-V3	8 (6.6)	0	1 (25.0)
	Extratrigeminal	3 (2.5)	0	1 (25.0)
Triggers	Speech	27 (22.1)	1 (9.1)	0
	Facial expressions	10 (8.2)	2 (18.2)	0
	Chewing	41 (33.6)	2 (18.2)	0
	Cold	9 (7.4)	0	0
	Touch	27 (22.1)	0	0
	Weather condition change	13 (10.7)	1 (9.1)	2 (50.0)
	Tooth brushing	9 (7.4)	1 (9.1)	0
	Jaw opening	19 (15.6)	0	0
	Face washing	8 (6.6)	0	0
	Hot beverages	3 (2.5)	0	0
	Swallowing	10 (8.2)	0	0
	Minimal strains and movements	4 (3.3)	0	1 (25.0)
	Coughing	1 (0.8)	0	0
Pain description	Stabbing	28 (23.0)	1 (9.1)	0
	Dull	2 (1.6)	2 (18.2)	2 (50.0)
	Shock-like	27 (22.1)	1 (10.0)	0
	Sharp	7 (5.7)	0	0
	Burning	2 (1.6)	3 (27.3)	0
	Throbbing	6 (4.9)	1 (9.1)	0
	Neuralgic	11 (9.0)	0	0
	Atypical	2 (1.6)	0	1 (25.0)
	Neuropathic	0	1 (9.1)	0

a-percentages calculated by total number of patients in a category. b-approximated to trigeminal divisions where possible. TN – trigeminal neuralgia, TNp –painful trigeminal neuropathy, pPIFP – presumed persistent idiopathic facial pain, V1-3 – trigeminal nerve divisions; Sums of % for certain groups are not necessarily 100. Missing data is reported in the Supplementary Data Table 1.

**Table 5.** Additional clinical characteristics of selected craniofacial pain categories.

Group, n (%^a^)		**TN, 122 (78.2)**	**TNp, 11 (7.1)**	**pPIFP, 4 (2.6)**
CAS	Nausea and vomiting	2 (1.6)	1 (9.1)	0
	Facial flushing	1 (0.8)	0	0
	Facial swelling	1 (0.8)	0	0
	Salivation	1 (0.8)	0	0
	Hypertension	1 (0.8)	0	0
	Lacrimation	10 (8.2)	1 (9.1)	0
	Photophobia	1 (0.8)	0	0
	Conjunctival hyperaemia	1 (0.8)	0	0
	Nasal congesition	0	0	0
	Rhinorrhea	4 (3.3)	1 (9.1)	0
SD	Allodynia	11 (9.0)	0	0
	Hyperesthesia	12 (9.8)	1 (9.1)	0
	Hypoaesthesia	6 (4.9)	2 (18.2)	0
	Dysesthesia	9 (7.4)	2 (18.2)	1 (25.0)
	Paresthesia	9 (7.4)	1 (9.1)	1 (25.0)

a-percentages calculated by total number of patients in a category; TN – trigeminal neuralgia, TNp –painful trigeminal neuropathy, pPIFP – presumed persistent idiopathic facial pain, CAS – Cranial autonomic symptoms, SD – sensory disturbances; Sums of % for certain groups are not necessarily 100. Missing data is reported in the Supplementary Data Table 1.

## Results

**Fig. 1:**
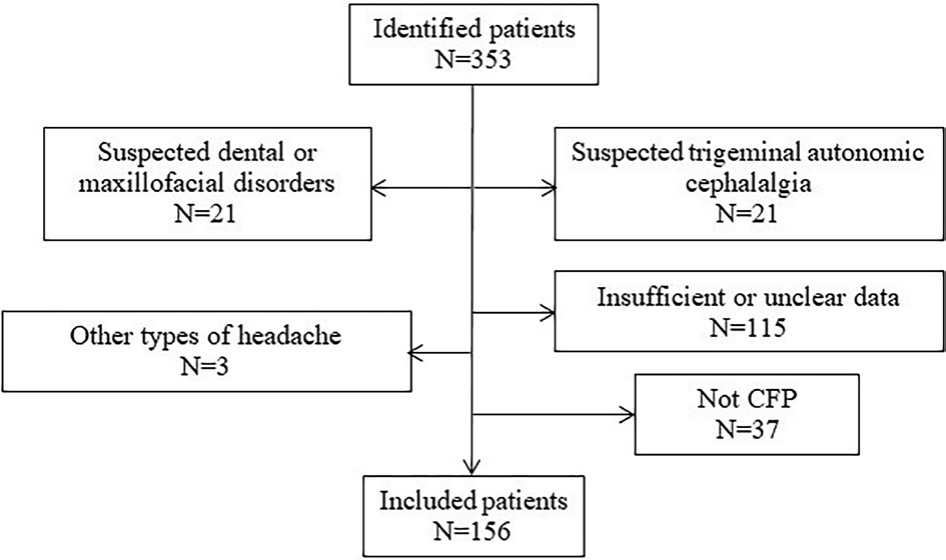
Flowchart of patients excluded from the study.

Among the 353 identified patients, 197 (55.8%) patients were excluded as shown in Fig. 1.

We included 156 patients and identified six types of CFPs (Table 1). Most patients (78.2%) had trigeminal neuralgia (TN), followed by painful trigeminal neuropathy (TNp, 7.1%), presumed occipital neuralgia (pON, 6.4%), presumed persistent idiopathic facial pain (pPIFP, 2.6%), glossopharyngeal neuralgia (GN, 2.6%), and painful nervus intermedius neuropathy (NINp, 2.6%). One patient had ipsilateral presumed TN and GN (pTN + pGN). None of the patients were hospitalized.

**Fig. 2:**
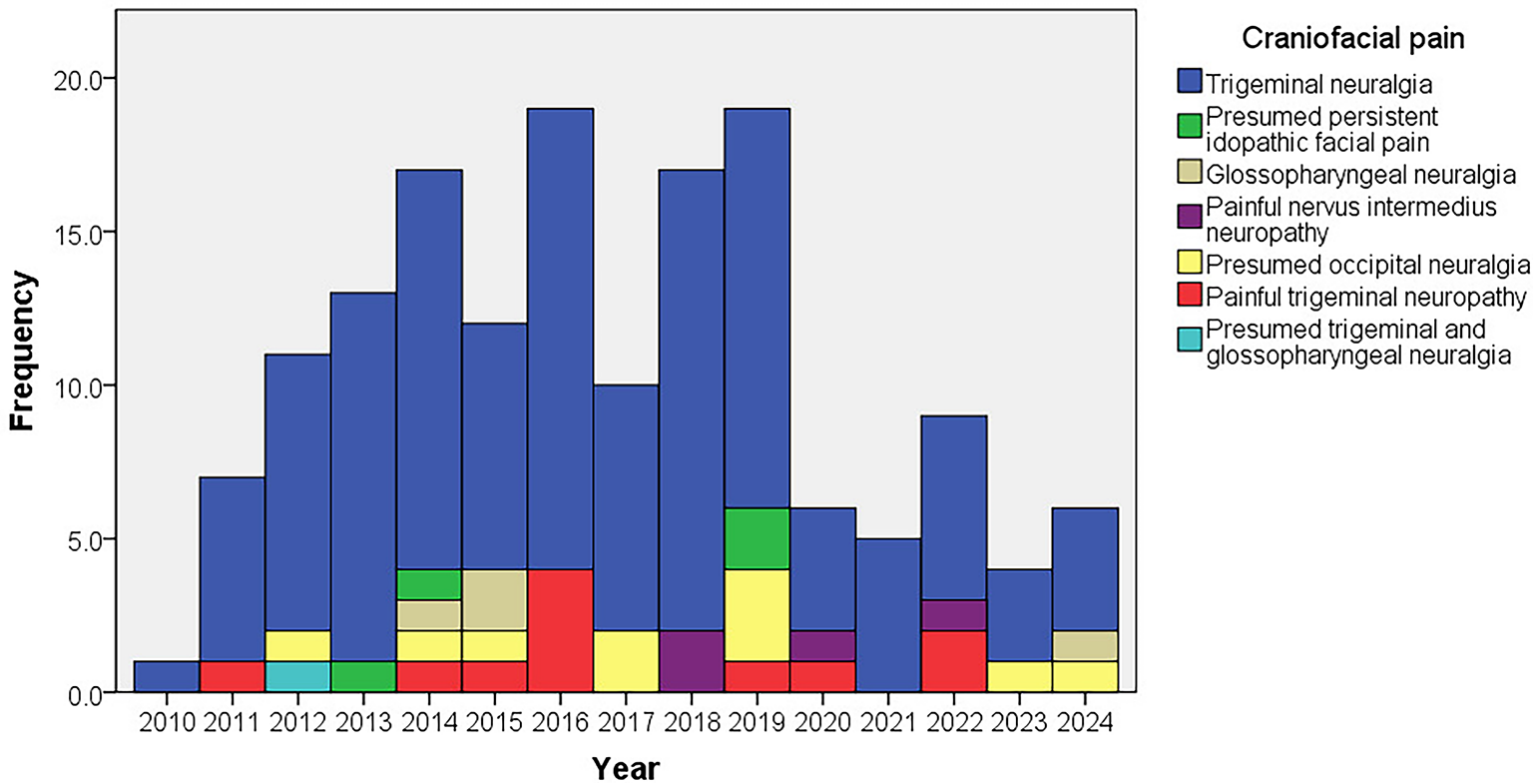
The distribution of patients over the years.

The distribution of patients over the years is shown in Fig. 2. Table 1. shows the demographic and clinical characteristics of the study population and CFPs. The patients usually presented in their seventh decade, except pPIFP (median 36 years). Females were disproportionately affected (69.2%), with a female-to-male ratio of 2.25:1. TNp was the only group with a slight male predominance (54.5%). In contrast to TN, other CFPs mainly presented in their first three months of pain (63.7 − 100%), with a monophasic-acute course (72.7 − 100%). The pain was usually right-sided (59.6%), except in GN (left side, 50.0%). It was very rarely bilateral (3.2%) and mild (1.9%). Approximately half of the patients (53.2%) reported different provoking factors, which were rare in TNp (18.2%) and absent in NINp (0%). These two groups had slightly more SD (45.5% and 50.0%, respectively) than the study group (34.6%). CAS were reported by patients who had TN (14.8%), TNp (18.2%), or pON (20.0%).

Treatment modalities used in the ED are shown in Table 2., and most frequently included parenteral nonopioid drugs (26.3%), mainly ketorolac (15.4%). Corticosteroids (4.5%) were occasionally given to patients with TN (4.9%) and pON (10.0%). Tramadol (8.3%) was used only for these CFPs (9.8% and 10% respectively).

**Table 6:** The frequency of sensory disturbances between patients based on history of invasive treatment.

Group n (%)		Sensory disturbances		Exact significance (2-sided)^*^
		Absent	Present	
Invasive treatment	No	69 (56.6)	33 (27.0)	0.606
	Yes	12 (9.8)	8 (6.6)	
	Total	81 (66.4)	41 (33.6)	

*Fisher’s exact test.

Medication prescribed at discharge is shown in Table 3 and Supplementary Data Table 2. The combined treatment (59.0%, Table 1) was centered on coanalgesics (87.8%, Table 3). Carbamazepine (67.3%) was the most frequently used drug, particularly for GN (100.0%) and TN (71.3%). Conversely, amitriptyline (TCA) (25.0%) was prescribed instead of carbamazepine (0%) in NINp. Patients with pPIFP were prescribed carbamazepine only in half of the cases, with the treatment centered on nonopioid analgesics (100.0%). Pregabalin was the second most used (14.1%) coanalgesic, particularly in TNp (36.4%) and pON (30.0%).

Tables 4 and 5. contain more details regarding pain distribution and descriptors, triggers, CAS, and SD for TN, TNp, and pPIFP. Additional characteristics for CFPs not shown in the tables will be provided in the following text.

A binomial test compared the observed number of CFP patients before (126/156) and after (30/156) the start of the COVID-19 pandemic against an expected distribution, revealing significantly fewer ED visits post-COVID-19 (*p* = 0.001, Supplementary Data Table 10). Similarly, for the TN subgroup (122/156), significantly fewer visits occurred post-COVID-19 (*p* = 0.001, Supplementary Data Table 10). There were no significant differences in CFP diagnoses and their frequencies based on their onset in relation to the COVID-19 outbreak (Supplementary Data Tables 3–9). Overall, there were no clinically meaningful differences in CFP characteristics between these two periods, and the comparison is severely limited by the small sample size (15 patients with onset in the post-COVID-19 period). These results are reported in the Supplementary Data (Supplementary Data Tables 3–9).

### Trigeminal neuralgia

The patients with defined etiology (24.6%) were divided into classical (9.0%), secondary (6.6%), and idiopathic (9.0%). Secondary TN was due to MS in 50.0%, followed by space-occupying lesions (25.0%), while 12.5% had MRI findings suggestive of vasculitis or demyelinating disorder, and 12.5% had postinflammatory trigeminal nerve enlargement.

There was a large group of etiologically undifferentiated TN (75.4%). This group included three patients (3.3%) with suspected secondary TN and six patients (6.5%) with suspected idiopathic TN.

The distribution of age at presentation (W = 0.951, *p* < 0.001) appears bimodal, with peaks around 35 and 75 years. The distribution of age at onset was also non-normal (W = 0.97, *p* = 0.003), but without a bimodal pattern.

The pain was almost exclusively unilateral (99.2%) and usually affected the V2 (25.4%), V3 (16.4%), or both branches (26.2%) (Tables 1 and 4). One-third of patients had different SDs (Tables 1 and 6). Twenty patients (16.4%) previously had invasive procedures (nerve blocks and lysis, neurovascular decompressive procedures, and radiosurgery), of which six had neurosurgical decompressive procedures (4.9%). There were no differences in SD frequency between patients based on a history of invasive procedures (*p* = 0.606, Table 6).

### Painful trigeminal neuropathy

Six patients had painful neuropathy attributed to the Varicella zoster virus (VZV) (54.5%), and 83.3% of them were males. Four patients (36.4%) had posttraumatic neuropathy caused by sinus surgery (2/4) or dental/endodontic treatment (2/4) and were exclusively female. One male patient (9.1%) had a painful TNp due to an invasive space-occupying lesion. The pain was mostly affecting the V1 branch (63.6%) in TNp, attributed to VZV and cancer, or V2 (36.4%) in posttraumatic TNp (Table 4). Patients with VZV were already prescribed antiviral medication by infectious disease, dermatology, or ophthalmology specialists.

### Presumed persistent idiopathic facial pain

Given that none had pain lasting more than three months, the diagnosis was labeled as presumed. The pain was poorly localized and approximated to trigeminal divisions. It affected the middle and lower areas of the face in 50.0%, but occasionally extended to the entire face (25.0%), or neck and shoulder (25.0%) (Table 4).

### Presumed occipital neuralgia

The diagnosis was classified as presumed since none of the patients received nerve blocks. The pain was described as throbbing (40.0%), shock-like (40.0%), stabbing, or pecking (10.0% each). The majority of patients reported pain in the minor occipital nerve division (45.5%), three in the major occipital nerve division (27.3%), one in the auriculotemporal region (9.1%), and one in an unspecified division (9.1%).

### Glossopharyngeal neuralgia

The pain was triggered by swallowing in 75.0% and described as stabbing, sharp, or pulsating (25.0%, respectively). Three patients (75.0%) reported pain in the oropharynx, while one (25.0%) had otic distribution. The etiology was unknown in 50.0%, while VZV and trauma after neck surgery were identified in the other cases (25.0% each).

### Painful nervus intermedius neuropathy

Neuropathy was due to VZV in all cases, and they had already been prescribed acyclovir. Ramsay Hunt syndrome (RHS) was diagnosed in three patients (75.0%) and was left-sided in one case (25.0%). Pain description was provided in one case (25.0%), classified as burning.

### Presumed trigeminal and glossopharyngeal neuralgia

The patient was an 86-year-old female who was examined within the first week of symptoms (Table 1). The pain was right-sided, severe, and throbbing. It was localized in the V2 and V3 areas with radiation to the pharynx. There were no triggers, CAS, or SD. The patient was treated with NSAIDs in the ED and recommended CBZ (Tables 1–3).

## Discussion

This 14-year retrospective study examines the neurological ED as an important and challenging setting for diagnosing and managing CFP. In contrast to prior research, which has predominantly explored outpatient populations, or certain CFP and headache subtypes in ED^1,2,8–12^, this study provides a unique focus on neuropathic CFP in an acute neurological setting, characterizing six distinct CFP categories. We identified a gap in acute treatment, which lacked guideline-supported rapid-onset options, relying instead on nonopioid and opioid analgesics. Furthermore, a notable reduction in CFP patient presentations to the neurological ED was observed post-COVID-19, likely attributable to external factors such as healthcare avoidance or access constraints. These findings offer new insights into CFP epidemiology and management, further elucidating its impact on emergency neurology.

Neurologists in the ED face significant challenges in managing CFP due to the need for a focused but thorough approach^1,3,4^. Generally, it is advisable to suspect secondary pathology until proven otherwise^5^. The incidence and prevalence of CFP are expected to increase with an aging population^10^. The typical age of onset for CFP occurs in the 50–60 s, although this varies by condition^1,3,5,13^. Our findings are consistent with prior studies, while the advanced age in the TNp and NINp groups could be due to VZV, as older patients are more susceptible to VZV infections, thereby elevating the overall age of these groups^1,5,14^.

The well-documented female predominance in most CFPs^5,10,15^ remains a complex issue, influenced by biological and psychosocial factors^15^. A slight male predominance in the TNp group, contrasting the typical findings^5^, is presumably due to patients with VZV. It may result from selection bias, given that the study focuses on patients reporting to the ED mainly due to pain exacerbation. Other possible psychosocial factors unique to ED patients require exploration.

### Trigeminal neuralgia

There is an underutilization of rapid-onset guideline-suggested treatment options for TN in emergency settings, coupled with a high rate of etiologically undefined cases due to diagnostic limitations, necessitating advanced imaging and updated treatment protocols.

Clinically established TN was the most common CFP with demographic and clinical characteristics similar to previous reports^3,5,8,16^. Age distribution showed a second peak in the thirties, suggesting possible underdiagnosed secondary trigeminal neuralgia^5,17^.

Extracting data on SD in TN was challenging due to reporting inconsistencies. Despite this, we noted a high (33.6%) prevalence of SD in patients with typical trigeminal pain, not attributable to previous surgical treatment. This finding is corroborated by previous studies focusing on subclinical SD in idiopathic or classic TN^18^, and SD found during examination of surgically naive patients^8^. While the absence of SD is no longer a diagnostic requirement for classic TN^5^, secondary pathology should still be considered given diagnostic constraints.

Distinguishing between TN and TAC can be difficult, especially when pain attacks are accompanied by CAS^4,5^. Although CAS are more prevalent in TN than previously assumed^8^, we would typically expect to find mild symptoms, mainly lacrimation^5,6^, as was the case in our patients (8.2%).

Despite TN being primarily a clinical diagnosis^5,6,16^, MRI is essential for ruling out secondary causes and subclassifying patients^3–6,17^. Given that imaging was limited to computerized tomography (CT), which is insufficient^5^, this prevented comprehensive subclassification of TN.

The first-line treatment for TN typically involves pharmacological options like CBZ, or oxcarbazepine, which has a better side-effect profile^3–5,17^. Contrary to chronic therapy, optimal management of acute exacerbations is unclear and variable^19^. The treatment would require medication with a rapid onset and routes of application other than oral due to triggering pain, anorexia, and vomiting^17,19^. European guidelines provide a weak recommendation for fosphenytoin and lidocaine^17^, while intravenous lacosamide may be another option with better safety than phenytoin^19,20^. This may necessitate hospitalization for rehydration and monitoring during treatment, as well as surgical evaluation^16,19^. Surgically, both neural and non-neural pathologies must be considered, including ossified ligaments, membranes, fascia, and variations in trigeminal porus types, as well as proximity to adjacent vascular or bony structures, which can contribute to nerve compression^21^. Given these intravenous medications are not available or not used in our neurological ED, neurologists administer nonopioid or opioid analgesics, despite a lack of evidence for their usefulness in TN^17,19,22^, The recommended therapy at discharge (CBZ) was more appropriate. Oxcarbazepine was not prescribed as it has only recently become available in our market.

### Painful trigeminal neuropathy

TNp is predominantly VZV related and requires an early intervention in the ED to prevent complications. The diagnosis and treatment were not significantly challenging for neurologists in the ED. The high prevalence of VZV goes in line with the patients’ advanced age and explains the involvement of V1^3,5,6^. The absence of tactile allodynia could be a consequence of insufficient reporting of examination findings. Negative sensory signs were present, possibly indicating an early stage of injury^23^, as most of the patients presented within three months. Treatments on discharge followed the recommendations for peripheral neuropathic pain^3,5^. Early treatment of acute infections with adequate doses of antiviral drugs and coanalgesics is important to prevent complications and the development of postherpetic neuralgia^3,5^.

### Presumed occipital neuralgia

pON is likely underdiagnosed in the ED due to the absence of confirmatory nerve blocks, revealing a significant diagnostic shortfall. As the third most frequent CFP in our study, pON is commonly encountered^5^, yet often confused with other headache disorders (migraine, tension-type, cervicogenic headache). According to ICHD-3, an occipital nerve block should be performed to diagnose and treat pON^5,6^. Since occipital nerve blocks are only performed by anesthesiologists at our institution and not in the ED, none of our patients received one during the examination. This limitation likely explains why pON appears underdiagnosed in our study compared to existing literature^24^, which reported a three times higher prevalence of ON (22–25%), especially in younger women^12^. While NSAIDs, paracetamol, opioids, and muscle relaxants can be used to treat acute pain and provide temporary relief, TCA and anticonvulsants are used for long-term therapy^5,25^. Our neurologists primarily prescribed CBZ and nonopioids, which only partially followed the recommendations.

### Presumed persistent idiopathic facial pain

pPIFP’s rarity and diagnostic complexity in the ED leads to guideline-deviant treatments, underscoring the need for multidisciplinary input. Although PIFP is not neuralgia, these cases highlight the difficulties of diagnosing CFP. The diagnosis requires the exclusion of dental and non-tooth-related orofacial pain, and other neuropathic facial pain (mostly TN)^3,4,26,27^. This was not possible in the ED because of time constraints and the limited multidisciplinary collaboration. Despite this, our patients with pPIFP seem to form a clinically distinct group, with primarily dull and „atypical pain“ in an approximated trigeminal distribution. Even considering various difficulties, it represents an underdiagnosed condition in the current study^26,28^. TCA and cognitive behavior therapy remain a mainstay of treatment^3,26,27^, which was not the case in our study. We assume this may stem from a misconception that PIFP is an “atypical TN” (based on CBZ recommendations)^27^, a lack of familiarity with treatment recommendations, and a diagnostic uncertainty that led neurologists to opt for NSAIDs until further evaluation could be done.

### Glossopharyngeal neuralgia

GN is more prevalent than previously reported, carries risks of misdiagnosis due to overlap with trigeminal neuralgia (TN), and responds well to carbamazepine (CBZ), though diagnostic and management challenges remain. GN is rare, more common in women, usually in their sixth decade^3,5,29^, and can present bilaterally in 25%, as in the current study. However, the prevalence was double (2.6%) compared to the literature (0.2 to 1.3%). Misdiagnosis is possible due to overlap with other CFPs, notably TN^4,29,30^. Nevertheless, treatment was straightforward, with CBZ being the most prescribed^3,5,29^. GN may also occur with TN up to 10–12%^4^. Wang et al. reported only 0.3–0.5% of such patients, totaling 14 cases during 12 years^31^. The diagnostic and therapeutic application of topical anesthesia in the pharynx^29^ was not used in the ED. Trigger factors, such as swallowing, are helpful to confirm the diagnosis but are not required^3,4,31^. This was the case in our pTN + pGN patient, which neurologists diagnosed primarily based on the distribution of pain without a typical trigger factor. Following an inadequate ED treatment with NSAIDs, the patient was appropriately recommended CBZ^5,29^. In cases of treatment failure and complex presentations, the involvement of a pain specialist or hospitalization could help determine the etiology and possible surgical strategies^4^.

### Painful nervus intermedius neuropathy

The cutaneous manifestations make the diagnosis of NINp attributed to VZV usually straightforward^14^. The incidence rises substantially with age, and people in their seventh and eighth decades of life are also more likely to develop RHS^32^, similar to the patients in the current study. The neurologists may have prescribed antidepressants due to existing or potential psychiatric comorbidities^3^.

### Impact of COVID-19

Our study finds a reduced number of CFP patients visiting the ED post-COVID-19, including the TN majority, which likely reflects fewer presentations rather than a decrease in disease incidence or exacerbations. Although a survey by the American Academy of Orofacial Pain suggested an increase in overall referrals for orofacial pain across settings^9^, only 6.3% (0–10.6% depending on the practice setting) reported an increase in neuropathic pain, while 47.7% reported number of referrals was still below pre-pandemic levels. A systematic review by Mitsikostas et al.^33^could not draw a formal conclusion on facial pain prevalence in relation to COVID-19 due to the lack of data. Based on our sample, CFP characteristics remained consistent regardless of whether symptoms first began before or after the pandemic. We suspect that external factors such as healthcare avoidance, reduced referrals, or ED access barriers during and after the pandemic drove the decline in visits, as was previously noted in other settings in the literature^34,35^. However, a longer period of post-COVID-19 follow-up is needed to establish if this decrease is sustained and to evaluate contributing factors. The small post-COVID-19 sample and retrospective design severely limit the statistical analysis. Additionally, patients with acute SARS-CoV-2 infection or in the immediate follow-up were referred to other centers. Prospective studies with larger cohorts, longer follow-up, and standardized data collection are needed to clarify how pandemics and other external factors influence ED utilization for CFP management.

### Role of the neurological ED

Many patients presented acutely, particularly those with rare conditions other than TN. The neurological ED in our region seems to represent the first or perhaps the second point of contact for these undifferentiated CFP patients. Establishing the correct diagnosis and providing adequate care in ED conditions can be challenging, often interrupted, and time-consuming. Considering that most patients did not require treatment during the examination, this may indicate a need to evaluate possible difficulties accessing the primary and higher outpatient healthcare levels.

To our knowledge, this is the first study focusing exclusively on CFP in a neurological ED, in contrast to prior work often limited to general emergency or outpatient settings, or specific conditions like trigeminal neuralgia and headaches. However, several limitations require addressing. As a retrospective study, clinical data were obtained from a non-standardized template, usually lacking a certain proportion of relevant information. Due to the retrospective design and limited documentation in the ED, confounding factors such as comorbidities and socioeconomic variables were not systematically assessed, though this aligns with the study’s focus on clinical description rather than causal analysis. The lack of follow-up after brief encounters in the ED prevents treatment evaluation. A limitation of this study is the small sample size and group sizes, including post-COVID-19 cases (*n* = 15 and *n* = 30), which constrained statistical analysis. There are diagnostic limitations, compromising the authors’ ability to compare the study population with the literature. The generalizability is constrained by the small sample size, single-center setting, reliance on clinical diagnoses, and unique demographic, systemic, and treatment characteristics. Additionally, this study relied on sex assigned in legal and medical records, which may not necessarily correspond to gender identity. Future research may benefit from including self-reported gender identity to better capture the complex relationship between sex, gender, and craniofacial pain.

Despite the acknowledged limitations, the extensive duration of this study, involving examinations by 25 neurologists, substantially enhances the clinical value and applicability of our data in the management of CFP in the neurological ED. Furthermore, these findings underscore the considerable challenges inherent in diagnosing and managing CFP within neurological emergency settings, thereby highlighting the critical need for ongoing research to establish evidence-based treatment protocols.

## Conclusion

The demanding clinical environment of the neurological ED complicates the accurate diagnosis and effective treatment of CFP. A notable decline in patient presentations to the neurological ED was observed post-COVID-19, likely attributable to external factors such as healthcare avoidance or access constraints. The typical patient was an elderly female with right-sided TN affecting the V2 and V3. Rare CFPs and their combinations can first present in the ED, positioning it as a critical initial or secondary point of contact for these patients. Neurologists generally followed the guidelines in most CFPs, yet updates are needed, particularly in optimizing the rapid-onset acute treatment. To enhance patient care, there is a need for institutional evidence-based guidelines for CFP management and uniform documentation based on ICHD-3 or ICOP.

## Electronic supplementary material

Below is the link to the electronic supplementary material.

Supplementary Material 1

## Data Availability

The datasets generated and/or analyzed during the current study are available from the corresponding author upon reasonable request.

## References

[1] Doneddu, P. E. et al. Neuropathic pain in the emergency setting: diagnosis and management. *J. Clin. Med.* **12**, 6028 (2023). 37762968 10.3390/jcm12186028 PMC10531819

[2] Rimmele, F. et al. Headache characteristics in the neurological emergency department: A retrospective study. *Front. Neurol.* **12**, (2021). 10.3389/fneur.2021.706074 PMC8416997 34489852

[3] Mullally, W. & Hall, K. Facial pain: evaluation and treatment in the emergency room. *Emerg. Med. Open. Access.* **06**, (2016).

[4] Van Deun, L. et al. Facial pain: A comprehensive review and proposal for a pragmatic diagnostic approach. *Eur. Neurol.* **83**, 5–16 (2020). 32222701 10.1159/000505727

[5] Tepper, S. J. Cranial neuralgias. *Contin Minneap. Minn.* **24**, 1157–1178 (2018). 10.1212/CON.0000000000000637 30074554

[6] Headache Classification Committee of the International Headache Society (IHS) The International Classification of Headache Disorders, 3rd edition. Cephalalgia 38, 1–211. (2018). 10.1177/0333102417738202 29368949

[7] International Classification of Orofacial Pain. 1st edition (ICOP). *Cephalalgia Int. J. Headache*. **40**, 129–221 (2020). 10.1177/0333102419893823 32103673

[8] Maarbjerg, S., Gozalov, A., Olesen, J. & Bendtsen, L. Trigeminal Neuralgia – A prospective systematic study of clinical characteristics in 158 patients. *Headache J. Head Face Pain*. **54**, 1574–1582 (2014). 10.1111/head.12441 25231219

[9] Yanez Regonesi, F., Kaspo, G. A. & Boggero, I. A. Moreno-Hay, I. The impact of the COVID-19 pandemic on orofacial pain practice. *J. Am. Dent. Assoc. 1939*. **154**, 266–271 (2023). 10.1016/j.adaj.2022.03.012 PMC9035375 35715264

[10] Häggman-Henrikson, B. et al. Increasing gender differences in the prevalence and chronification of orofacial pain in the population. *Pain* **161**, 1768–1775 (2020). 32701837 10.1097/j.pain.0000000000001872 PMC7365674

[11] Ziegeler, C., Brauns, G. & May, A. Characteristics and natural disease history of persistent idiopathic facial pain, trigeminal neuralgia, and neuropathic facial pain. *Headache* **61**, 1441–1451 (2021). 34618363 10.1111/head.14212

[12] Mathew, P. G., Najib, U., Khaled, S. & Krel, R. Prevalence of occipital neuralgia at a community Hospital-based headache clinic. *Neurol. Clin. Pract.* **11**, 6–12 (2021). 33968466 10.1212/CPJ.0000000000000789 PMC8101323

[13] Macfarlane, T. V., Beasley, M. & Macfarlane, G. J. Self-Reported facial pain in UK biobank study: prevalence and associated factors. *J. Oral Maxillofac. Res.* **5**, e2 (2014). 25386229 10.5037/jomr.2014.5302 PMC4219861

[14] O’Neill, F., Nurmikko, T. & Sommer, C. Other facial neuralgias. *Cephalalgia* **37**, 658–669 (2017). 28133989 10.1177/0333102417689995

[15] Samulowitz, A., Gremyr, I., Eriksson, E. & Hensing, G. Brave Men and Emotional Women: A Theory-Guided Literature Review on Gender Bias in Health Care and Gendered Norms towards Patients with Chronic Pain. *Pain Res. Manag.* 6358624 (2018). (2018). 10.1155/2018/6358624 PMC5845507 29682130

[16] Lambru, G., Zakrzewska, J. & Matharu, M. Trigeminal neuralgia: a practical guide. *Pract. Neurol.* **21**, 392–402 (2021). 34108244 10.1136/practneurol-2020-002782 PMC8461413

[17] Bendtsen, L. et al. European academy of neurology guideline on trigeminal neuralgia. *Eur. J. Neurol.* **26**, 831–849 (2019). 30860637 10.1111/ene.13950

[18] Araya, E. I., Claudino, R. F., Piovesan, E. J. & Chichorro, J. G. Trigeminal neuralgia: basic and clinical aspects. *Curr. Neuropharmacol.* **18**, 109–119 (2020). 31608834 10.2174/1570159X17666191010094350 PMC7324879

[19] Chow, A., Haider, I., Athanaselos, A. & Patel, M. In-Hospital management of acute trigeminal neuralgia pain crises. *Clin. Med. Res.* **22**, 215–221 (2024). 39993829 10.3121/cmr.2024.1945 PMC11849969

[20] Muñoz-Vendrell, A., Teixidor, S., Sala-Padró, J., Campoy, S. & Huerta-Villanueva, M. Intravenous lacosamide and phenytoin for the treatment of acute exacerbations of trigeminal neuralgia: A retrospective analysis of 144 cases. *Cephalalgia Int. J. Headache*. **42**, 1031–1038 (2022). 10.1177/03331024221092435 PMC9442778 35469475

[21] Ogut, E., Armagan, K. & Barut, C. Reappraisal of the types of trigeminal porus and importance in surgical applications. *Surg. Radiol. Anat.* **43**, 1169–1178 (2021). 33399922 10.1007/s00276-020-02651-z

[22] Zakrzewska, J. M. et al. Characterizing treatment utilization patterns for trigeminal neuralgia in the united States. *Clin. J. Pain*. **34**, 691–699 (2018). 29443722 10.1097/AJP.0000000000000595

[23] Droguett Tidy, C. & Lolas Millard, J. Labbé Martínez, C. Painful traumatic trigeminal neuropathy. Diagnosis and treatment: about two clinical cases. *Rev. Esp. Cir. Oral Maxilofac*. **43**, 109–116 (2021).

[24] Molina, O. F. et al. Occipital neuralgia as a true neuropathic pain. *Rev. Neurociências*. **22**, 242–248 (2014).

[25] Choi, I. & Jeon, S. R. Neuralgias of the head: occipital neuralgia. *J. Korean Med. Sci.* **31**, 479–488 (2016). 27051229 10.3346/jkms.2016.31.4.479 PMC4810328

[26] Benoliel, R. & Gaul, C. Persistent idiopathic facial pain. *Cephalalgia* **37**, 680–691 (2017). 28425324 10.1177/0333102417706349

[27] Gerwin, R. & Chronic Facial, P. Trigeminal neuralgia, persistent idiopathic facial pain, and myofascial pain Syndrome-An Evidence-Based narrative review and etiological hypothesis. *Int. J. Environ. Res. Public. Health*. **17**, 7012 (2020). 32992770 10.3390/ijerph17197012 PMC7579138

[28] Zakrzewska, J. M. Chronic/Persistent idiopathic facial pain. *Neurosurg. Clin. N Am.* **27**, 345–351 (2016). 27325001 10.1016/j.nec.2016.02.012

[29] Shah, R. J. & Padalia, D. Glossopharyngeal neuralgia. In *StatPearls* (StatPearls Publishing, 2024). 31082085

[30] Wu, L. et al. Case report: trigeminal neuralgia misdiagnosed as glossopharyngeal neuralgia. *Front. Neurol.* **14**, 1079914 (2023). 36741284 10.3389/fneur.2023.1079914 PMC9892896

[31] Wang, X., Meng, D., Wang, L. & Chen, G. The clinical characteristics and surgical treatment of glossopharyngeal neuralgia with pain radiating to the innervated area of the trigeminal nerve. *J. Oral Maxillofac. Surg.* **79**, 786e1–786e8 (2021). 10.1016/j.joms.2020.11.034 33387474

[32] Goswami, Y. & Gaurkar, S. S. Ramsay Hunt syndrome: an introduction, signs and symptoms, and treatment. *Cureus*10.7759/cureus.33688 (2023). 36793818 10.7759/cureus.33688 PMC9925029

[33] Mitsikostas, D. D. D. et al. Headaches and facial pain attributed to SARS-CoV-2 infection and vaccination: a systematic review. *Eur. J. Neurol.* **31**, e16251 (2024). 38415282 10.1111/ene.16251 PMC11235838

[34] Maretta, M., Škorvánek, M., Jurková, V., Leško, N. & Gdovinová, Z. Impact of the COVID-19 outbreak on neurological consultation in an emergency department. *Acta Neurol. Belg.* **123**, 295–297 (2023). 35034334 10.1007/s13760-021-01852-6 PMC8761110

[35] Flamm, A., Lee, A. & Mencl, F. COVID-19: A comprehensive analysis of the pandemic’s effect on an emergency department. *Disaster Med. Public. Health Prep*. 1–4. 10.1017/dmp.2021.182 (2021). 10.1017/dmp.2021.182 PMC8314046 34099096

