# Supplementary material for: Fourteen year retrospective study of craniofacial pain in a neurological emergency department
Source: Sci Rep. 2025 Sep 30;15:33775. doi: 10.1038/s41598-025-01246-1 (PMC12484931; doi:10.1038/s41598-025-01246-1)
Supplement: Supplementary file 1 — Supplementary Material 1 [file 41598_2025_1246_MOESM1_ESM.docx]

**Fourteen Year Retrospective Study of Craniofacial Pain in Neurological Emergency Department**

Aleksandra Lučić MD, PhD*^1,2^, Zlatko Božić MD^1,2^, Aleksandar Kopitović MD, PhD^1,2^, Sanela Popović MD^1,2^, Dane Krtinić MD, PhD^3,4^ and Igor Petrušić MD, PhD^5^

^a^Neurology clinic, University Clinical Center of Vojvodina, Hajduk Veljkova 1, 21137 Novi Sad, Serbia

^b^Faculty of Medicine, University of Novi Sad, Hajduk Veljkova 3, 21137 Novi Sad, Serbia

^c^Department for pharmacology and toxicology, Faculty of Medicine, University of Niš, Blvd. Dr Zorana Djindjica 81, 18108 Niš, Serbia

^d^Clinic for Oncology, University Clinical Center Niš, Blvd. Dr Zorana Đinđića 48, 18108 Niš, Serbia

^e^Laboratory for Advanced Analysis of Neuroimages, Faculty of Physical Chemistry, University of Belgrade, Studentski trg 12-16, 11158 Belgrade 118, Serbia

# SUPPLEMENTARY DATA

**Supplementary Data Table 1.** Missing data.

| **Group, n (%^a^)** | **Total**  **94^b^ (60.3)** | **TN**  **76 ^b^ (62.3)** | **TNp**  **6 ^b^ (54.5)** | **pON**  **5 ^b^ (50.0)** | **pPIFP**  **1 ^b^ (25.0)** | **GN**  **2 ^b^ (50.0)** | **NINp**  **4 ^b^ (100.0)** | **pTN+pGN 0** |
| --- | --- | --- | --- | --- | --- | --- | --- | --- |
| **Age at Onset** | 14 (9.0) | 12 (9.8) | 1 (9.1) | 1 (10.0) | 0 | 0 | 0 | 0 |
| **Duration** | 8 (5.1) | 5 (4.1) | 1 (9.1) | 2 (20.0.) | 0 | 0 | 0 | 0 |
| **Affected side** | 2 (1.3) | 1 (0.8) | 0 | 1 (10.0.) | 0 | 0 | 0 | 0 |
| **Disribution** | 5 (3.2) | 5 (4.1) | 0 | 0 | 0 | 0 | 0 | 0 |
| **Pain description** | 44 (28.2) | 37 (30.3) | 2 (18.2) | 0 | 1 (25.0) | 1 (25.0) | 3 (75.0.) | 0 |
| **Intensity** | 46 (29.5) | 35 (28.7) | 2 (18.2) | 4 (40.0.) | 1 (25.0) | 2 (50.0) | 2 (50.0) | 0 |
| **Trigger** | 32 (20.5) | 25 (20.5) | 5 (45.5) | 0 | 0 | 0 | 2 (50.0) | 0 |
| **CAS** | 3 (1.9) | 3 (2.5) | 0 | 0 | 0 | 0 | 0 | 0 |
| **Treatment at Discharge** | 4**^*^** (2.6) | 3^*^ (2.5) | 1^*^ (9.1) | 0 | 0 | 0 | 1^*^(25.0) | 0 |
| **COVID-19 period** | 1 (0.6) | 1 (0.8) | 0 | 0 | 0 | 0 | 0 | 0 |

a-percentages calculated by total number of patients in a selected study group; b – at least one missing data point; TN – trigeminal neuralgia, TNp – painful trigeminal neuropathy, pON – presumed occipital neuralgia, pPIFP – presumed persistent idiopathic facial pain, GN – glossopharyngeal neuralgia; NINp – painful nervus intermedius neuropathy; pTN+pGN – presumed concomitant trigeminal and glossopharyngeal neuralgia; CAS – Cranial autonomyc symptoms; Sums of for certain grups are not necessarily 100. * Treatment:: One patient with TN was recommended to continue the existing treatment regimen, which was not written in the report. That patient has missing data for all medication. Three patients (singular patients with TN, TNp and NINp) were recommended nonspecific nonopioid analgesics. One patient with TN has missing data about CAMs

**Supplementary Table 2.** Treatment at discharge – nonopioids

| **Group, n (%^a^)** | **Total, 156** | **TN, 122 (78.2)** | **TNp, 11 (7.1)** | **pON, 10 (6.4)** | **pPIFP, 4 (2.6)** | **GN, 4 (2.6)** | **NINp, 4 (2.6)** | **pTN+pGN, 1 (0.6)** |
| --- | --- | --- | --- | --- | --- | --- | --- | --- |
| **Nonopioids** | 56 (35.9) | **38**^‡^ (**31.1**‡) | 6 (54.5) | 5 (50.0) | 4 (100.0) | 1 (25.0) | 2 (50.0) | 0 |
| Paracetamol | 31 (19.9) | 21 (17.2) | 3 (27.3) | 3 (30.0) | 3 (75.0) | 1 (25.0) | 0 | 0 |
| Paracetamol and  Ibuprofen (single pill) | 7 (4.5) | 5 (4.1) | 1 (9.1) | 1 (10.0) | 0 | 0 | 0 | 0 |
| Ibuprofen | 9 (5.8) | 4 (3.3) | 0 | 3 (30.0) | 2 (50.0) | 0 | 0 | 0 |
| Dexketoprofen | 1 (0.6) | 0 | 0 | 0 | 1 (25.0 | 0 | 0 | 0 |
| Naproxen | 2 (1.3) | 1 (0.8) | 0 | 1 (10.0) | 0 | 0 | 0 | 0 |
| Diclofenac | 2 (1.3) | 2 (1.6) | 0 | 0 | 0 | 0 | 0 | 0 |
| Ketorolac | 9 (5.8) | 8 (6.6) | 0 | 0 | 0 | 0 | 1 (25.0) | 0 |
| Metamizole | 4 (2.6) | 2 (1.6) | 1 (9.1) | 1 (10.0) | 0 | 0 | 0 | 0 |

a-percentages calculated by total number of patients in a selected group. TN – trigeminal neuralgia, TNp –painful trigeminal neuropathy, pON –presumed occipital neuralgia, pPIFP – presumed persistent idiopathic facial pain, GN – glossopharyngeal neuralgia; NINp – painful intermedius nerve neuropathy; pTN+pGN – presumed concomitant trigeminal and glossopharyngeal neuralgia; Sums of % for certain groups are not necessarily 100. Missing data is reported in the Supplementary Data Table 1.

## Difference in craniofacial pain profiles before and after the beginning of the Covid-19 pandemic

Patients were grouped based on whether the disease onset was before or after the beginning of the Covid-19 pandemic in Serbia. Association between categorical variables was analyzed using the Pearson Chi square test. We used the Fisher’s exact test for 2x2 contingency tables. The difference in age distribution between the groups was analyzed using the Mann-Whitney U test. There were no significant differences in CFP types and their frequencies before and after COVID-19 outbreak (**χ^2^** (6)=7.276, p=0.296;**Fisher’s test,** p=0.099 Supplementary Data Tables 3 and 4).

**Supplementary Data Table 3.** Craniofacial pain type frequencies before and after COVID-19 outbreak.

| **Group** | | **COVID-19 Period** | | | | **Total** | | | **Pearson Chi-Square** | | |
| --- | --- | --- | --- | --- | --- | --- | --- | --- | --- | --- | --- |
|  |  | **pre** | | **post** | |  |  |  |  |  |  |
|  |  | **n** | **Expected** | **n** | **Expected** | **n** | **Expected** | **%** | **χ^2^** | **df** | **p value** |
| **CFP** | **TN** | 112_a_ | 109.3 | 9_a_ | 11.7 | 121 | 121.0 | 78.1 | 7.276 | 6 | 0.296 |
|  | **TNp** | 8_a_ | 9.9 | 3_a_ | 1.1 | 11 | 11.0 | 7.1 |  |  |  |
|  | **pON** | 9_a_ | 9.0 | 1_a_ | 1.0 | 10 | 10.0 | 6.5 |  |  |  |
|  | **pPIFP** | 4_a_ | 3.6 | 0_a_ | 0.4 | 4 | 4.0 | 2.6 |  |  |  |
|  | **GN** | 3_a_ | 3.6 | 1_a_ | 0.4 | 4 | 4.0 | 2.6 |  |  |  |
|  | **NINp** | 3_a_ | 3.6 | 1_a_ | 0.4 | 4 | 4.0 | 2.6 |  |  |  |
|  | **TN+GN** | 1_a_ | 0.9 | 0_a_ | 0.1 | 1 | 1.0 | 0.6 |  |  |  |
| **Total** | | 140 | 140.0 | 15 | 15.0 | 155 | 155.0 | 100.0 |  |  |  |

Each subscript letter denotes a subset of craniofacial pain categories whose column proportions do not differ significantly from each other at the 0.05 level. χ^2^ –test score, df – degrees of freedom; CFP – craniofacial pain types, TN – trigeminal neuralgia, TNp – painful trigeminal neuropathy, pON – presumed occipital neuralgia, pPIFP – presumed persistent idiopathic facial pain, GN – glossopharyngeal neuralgia; NINp – painful nervus intermedius neuropathy; pTN+pGN – presumed concomitant trigeminal and glossopharyngeal neuralgia. Missing data is reported in the Supplementary Data Table 1.

**Supplementary Data Table 4.** Comparison of trigeminal neuralgia frequency with other craniofacial pain types before and after COVID-19 outbreak

| **Group** | | **COVID-19 period** | | | | **Total** | | | **Fishers Exact test** |
| --- | --- | --- | --- | --- | --- | --- | --- | --- | --- |
|  |  | **pre** | | **post** | |  |  |  |  |
|  |  | **n** | **Expected** | **n** | **Expected** | **n** | **Expected** | **%** |  |
| CFP | TN | 112_a_ | 109.3 | 9_a_ | 11.7 | 121 | 121.0 | 78.1 | 0.099 |
|  | other | 28_a_ | 30.7 | 6_a_ | 3.3 | 34 | 34.0 | 21.9 |  |
| Total | | 140 | 140.0 | 15 | 15.0 | 155 | 155.0 | 100.0 |  |

Each subscript letter denotes a subset of craniofacial pain categories whose column proportions do not differ significantly from each other at the 0.05 level. CFP – craniofacial pain types, TN – trigeminal neuralgia. Missing data is reported in the Supplementary Data Table 1.

The duration of CFPs in the post COVID-19 period was mostly under three months, and there was significantly less patients with the condition lasting more than one year (**χ^2^** (3)= 10.496, p=0.015, Supplementary Data Table 5). However, the entire post COVID group is small, with only 15 patients, and the significance is driven by a single patient, which violates test assumptions. The result regarding difference in course, indicating significantly less patents with relapsing-remitting and chronic course (**χ^2^** (2)= 7.612, p=0.022, Supplementary Data Table 5), behaves in a similar fashion as these variables are connected. Similarly, small cell counts produce the significant difference in the affected side (**χ^2^** (2)= 6.744, p=0.035, Supplementary Data Table 5), which offers littleor no value. More data must be collected to draw a clinically meaningful conclusion (Supplementary Data Table 5).

Overall, there were no clinically meaningful differences in characteristics of patients with CFP between these two periods. Tests with significant results were of limited value as the results were driven by cells with small counts violating test assumptions. Therefore we decided against conducting further analysis for TN. Other CFP groups were also not large enough to conduct statistical analysis. The results are shown in the Supplementary Data Tables 5-9.

**Supplementary Data Table 5.** Demographic and clinical characteristics of the study population before and after COVID-19 outbreak.

| **Group** | | **COVID-19 period** | | | | **Test** | | | | | | | |
| --- | --- | --- | --- | --- | --- | --- | --- | --- | --- | --- | --- | --- | --- |
|  |  | **pre** | | **post** | |  |  |  |  |  |  |  |  |
|  |  | **n** | **%^a^** | **n** | **%^a^** |  |  |  |  |  |  |  |  |
| **Age at presentation** | Median, IQR | 61.5, 29 | | 57.0, 33 | | **Mann Whitney U test** | | | | | | | |
|  |  |  |  |  |  | **U** | **W** | | | **Z** | | | **p** |
|  |  |  |  |  |  | 1025.0 | 10895.0 | | | -0.151 | | | 0.880 |
| **Age at onset** | Median, IQR | 56.0, 30 | | 57.0, 33 | | 812.0 | 8940.0 | | | -0.933 | | | 0.351 |
| **Sex** | male | 42 | 30.0 | 5 | 33.3 | **Fisher’s exact test** | | | | | | | |
|  | female | 98 | 70.0 | 10 | 66.7 | 0.774 | | | | | | | |
| **Duration** | < 1 week | 21 | 15.0 | 5 | 33.3 | **Chi square** | | | | | | | |
|  | 1 week – 3 months | 30 | 21.4 | 6 | 40.0 | **χ^2^** | | | **df** | | **p** | | |
|  | 3 months – 1 year | 15 | 10.7 | 3 | 20.0 | 10.496 | | | 3 | | **0.015** | | |
|  | > 1 year | 67 | 47.9 | 1 | 6.7 |  |  |  |  |  |  |  |  |
| **Course** | monophasic - acute | 53 | 37.9 | 11 | 73.3 | 7.612 | | | 2 | | **0.022** | | |
|  | relapsing - remitting | 65 | 46.4 | 2 | 13.3 |  |  |  |  |  |  |  |  |
|  | chronic | 22 | 15.7 | 2 | 13.3 |  |  |  |  |  |  |  |  |
| **Affected Side** | right | 87 | 62.1 | 6 | 40.0 | 6.744 | | | 2 | | **0.034** | | |
|  | left | 49 | 35.0 | 6 | 40.0 |  |  |  |  |  |  |  |  |
|  | both | 3 | 2.1 | 2 | 13.3 |  |  |  |  |  |  |  |  |
| **Pain intensity** | mild | 3 | 2.1 | 0 | 0.0 | 4.036 | | | 2 | | 0.133 | | |
|  | moderate | 36 | 25.7 | 1 | 6.7 |  |  |  |  |  |  |  |  |
|  | severe | 59 | 42.1 | 10 | 66.7 |  |  |  |  |  |  |  |  |
| **Trigger** | present | 76 | 54.3 | 7 | 46.7 | **Fisher’s exact test** | | | | | | | |
|  |  |  |  |  |  | 0.081 | | | | | | | |
| **CAS** | present | 20 | 14.3 | 2 | 13.3 | 1.000 | | | | | | | |
| **SD** | present | 49 | 35.0 | 4 | 26.7 | 0.582 | | | | | | | |
| **Treated during visit** | no therapy | 3 | 2.1 | 1 | 6.7 | **Chi square test** | | | | | | | |
|  | monoterapy | 54 | 38.6 | 5 | 33.3 | **χ^2^** | | **df** | | | | **p** | |
|  | polytherapy | 82 | 58.6 | 9 | 60.0 | 1.169 | | 2 | | | | 0.557 | |
| **Therapy at discharge** | no therapy | 90 | 64.3 | 11 | 73.3 | 0.733 | | 2 | | | | 0.693 | |
|  | monoterapy | 32 | 22.9 | 2 | 13.3 |  |  |  |  |  |  |  |  |
|  | polytherapy | 18 | 12.9 | 2 | 13.3 |  |  |  |  |  |  |  |  |

a-percentages calculated by total number of patients in a selected group; IQR – interquartile range; U – test score; W – Wilcoxon Signed Rank Test; Z – z score; χ^2^ –test score, df – degrees of freedom; CAS – cranial autonomic symptoms, SD – sensory disturbances; Sums of % for certain groups are not necessarily 100. Missing data is reported in the Supplementary Data Table 1.

**Supplementary Data Table 6.** Demographic and clinical characteristics of craniofacial pain types before and after the COVID-19 outbreak.

| **Group** | | | | **Craniofacial pain type** | | | | | | | | | | | | | |
| --- | --- | --- | --- | --- | --- | --- | --- | --- | --- | --- | --- | --- | --- | --- | --- | --- | --- |
|  |  |  |  | **TN** | | **TNp** | | **pON** | | **pPIFP** | | **GN** | | **NINp** | | **pTN+pGN** | |
|  |  |  |  | **n** | **%^a^** | **n** | **%^a^** | **n** | **%^a^** | **n** | **%^a^** | **n** | **%^a^** | **n** | **%^a^** | **n** | **%^a^** |
| **COV-19** | **pre** | **Age at presentation** | Median, IQR | 62, 42-72 | | 72, 60-74 | | 62, 46-68 | | 36, 33-42 | | 52, 50-70 | | 73, 61-83 | | 86 | |
|  |  | **Age at onset** | Median IQR | 56, 38-67 | | 72, 59-75 | | 57, 46-69 | | 36, 33-42 | | 52, 50-64 | | 73, 61-83 | | 86 | |
|  |  | **Sex** | female | 76 | 67.9 | 5 | 62.5 | 7 | 77.8 | 4 | 100.0 | 3 | 100.0 | 2 | 66.7 | 1 | 100.0 |
|  |  | **Duration** | < 1 week | 13 | 11.6 | 1 | 12.5 | 3 | 33.3 | 2 | 50.0 | 0 | 0.0 | 1 | 33.3 | 1 | 100.0 |
|  |  |  | 1 week – 3 months | 17 | 15.2 | 4 | 50.0 | 3 | 33.3 | 2 | 50.0 | 2 | 66.7 | 2 | 66.7 | 0 | 0.0 |
|  |  |  | 3 months – 1 year | 15 | 13.4 | 0 | 0.0 | 0 | 0.0 | 0 | 0.0 | 0 | 0.0 | 0 | 0.0 | 0 | 0.0 |
|  |  |  | > 1 year | 63 | 56.3 | 2 | 25.0 | 1 | 11.1 | 0 | 0.0 | 1 | 33.3 | 0 | 0.0 | 0 | 0.0 |
|  |  | **Course** | monophasic - acute | 31 | 27.7 | 6 | 75.0 | 6 | 66.7 | 4 | 100.0 | 2 | 66.7 | 3 | 100.0 | 1 | 100.0 |
|  |  |  | relapsing - remitting | 61 | 54.5 | 1 | 12.5 | 3 | 33.3 | 0 | 0.0 | 0 | 0.0 | 0 | 0.0 | 0 | 0.0 |
|  |  |  | chronic | 20 | 17.9 | 1 | 12.5 | 0 | 0.0 | 0 | 0.0 | 1 | 33.3 | 0 | 0.0 | 0 | 0.0 |
|  |  | **Affected Side** | right | 69 | 61.6 | 6 | 75.0 | 6 | 66.7 | 2 | 50.0 | 0 | 0.0 | 3 | 100.0 | 1 | 100.0 |
|  |  |  | left | 42 | 37.5 | 2 | 25.0 | 2 | 22.2 | 1 | 25.0 | 2 | 66.7 | 0 | 0.0 | 0 | 0.0 |
|  |  |  | both | 1 | 0.9 | 0 | 0.0 | 0 | 0.0 | 1 | 25.0 | 1 | 33.3 | 0 | 0.0 | 0 | 0.0 |
|  |  | **Pain Intensity** | mild | 3 | 2.7 | 0 | 0.0 | 0 | 0.0 | 0 | 0.0 | 0 | 0.0 | 0 | 0.0 | 0 | 0.0 |
|  |  |  | moderate | 24 | 21.4 | 6 | 75.0 | 2 | 22.2 | 2 | 50.0 | 2 | 66.7 | 0 | 0.0 | 0 | 0.0 |
|  |  |  | severe | 51 | 45.5 | 1 | 12.5 | 3 | 33.3 | 1 | 25.0 | 0 | 0.0 | 2 | 66.7 | 1 | 100.0 |
|  |  | **Trigger** | present | 66 | 58.9 | 2 | 25.0 | 4 | 44.4 | 2 | 50.0 | 2 | 66.7 | 0 | 0.0 | 0 | 0.0 |
|  |  | **CAS** | present | 16 | 14.3 | 2 | 25.0 | 2 | 22.2 | 0 | 0.0 | 0 | 0.0 | 0 | 0.0 | 0 | 0.0 |
|  |  | **SD** | present | 39 | 34.8 | 3 | 37.5 | 4 | 44.4 | 1 | 25.0 | 1 | 33.3 | 1 | 33.3 | 0 | 0.0 |
|  |  | **Treated during visit** | no therapy | 73 | 65.2 | 4 | 50.0 | 5 | 55.6 | 3 | 75.0 | 3 | 100.0 | 2 | 66.7 | 0 | 0.0 |
|  |  |  | monoterapy | 24 | 21.4 | 3 | 37.5 | 2 | 22.2 | 1 | 25.0 | 0 | 0.0 | 1 | 33.3 | 1 | 100.0 |
|  |  |  | polytherapy | 15 | 13.4 | 1 | 12.5 | 2 | 22.2 | 0 | 0.0 | 0 | 0.0 | 0 | 0.0 | 0 | 0.0 |
|  |  | **Therapy at discharge** | no therapy | 2 | 1.8 | 0 | 0.0 | 0 | 0.0 | 0 | 0.0 | 0 | 0.0 | 1 | 33.3 | 0 | 0.0 |
|  |  |  | monoterapy | 47 | 42.0 | 1 | 12.5 | 4 | 44.4 | 0 | 0.0 | 1 | 33.3 | 0 | 0.0 | 1 | 100.0 |
|  |  |  | polytherapy | 62 | 55.4 | 7 | 87.5 | 5 | 55.6 | 4 | 100.0 | 2 | 66.7 | 2 | 66.7 | 0 | 0.0 |
|  | **post** | **Age at presentation** | Median, IQR | 57, 39-71 | | 45, 41-81 | | 74 | | / | | 86 | | 56 | | / | |
|  |  | **Age at onset** | Median IQR | 57, 39-68 | | 45, 41-81 | | 74 | | / | | 86 | | 56 | | / | |
|  |  | **Sex** | female | 8 | 88.9 | 0 | 0.0 | 1 | 100.0 | 0 | 0.0 | 0 | 0.0 | 1 | 100.0 | 0 | 0.0 |
|  |  | **Duration** | < 1 week | 3 | 33.3 | 1 | 33.3 | 1 | 100.0 | 0 | 0.0 | 0 | 0.0 | 0 | 0.0 | 0 | 0.0 |
|  |  |  | 1 week – 3 months | 3 | 33.3 | 1 | 33.3 | 0 | 0.0 | 0 | 0.0 | 1 | 100.0 | 1 | 100.0 | 0 | 0.0 |
|  |  |  | 3 months – 1 year | 2 | 22.2 | 1 | 33.3 | 0 | 0.0 | 0 | 0.0 | 0 | 0.0 | 0 | 0.0 | 0 | 0.0 |
|  |  |  | > 1 year | 1 | 11.1 | 0 | 0.0 | 0 | 0.0 | 0 | 0.0 | 0 | 0.0 | 0 | 0.0 | 0 | 0.0 |
|  |  | **Course** | monophasic - acute | 6 | 66.7 | 2 | 66.7 | 1 | 100.0 | 0 | 0.0 | 1 | 100.0 | 1 | 100.0 | 0 | 0.0 |
|  |  |  | relapsing - remitting | 2 | 22.2 | 0 | 0.0 | 0 | 0.0 | 0 | 0.0 | 0 | 0.0 | 0 | 0.0 | 0 | 0.0 |
|  |  |  | chronic | 1 | 11.1 | 1 | 33.3 | 0 | 0.0 | 0 | 0.0 | 0 | 0.0 | 0 | 0.0 | 0 | 0.0 |
|  |  | **Affected Side** | right | 4 | 44.4 | 0 | 0.0 | 1 | 100.0 | 0 | 0.0 | 1 | 100.0 | 0 | 0.0 | 0 | 0.0 |
|  |  |  | left | 4 | 44.4 | 1 | 33.3 | 0 | 0.0 | 0 | 0.0 | 0 | 0.0 | 1 | 100.0 | 0 | 0.0 |
|  |  |  | both | 0 | 0.0 | 2 | 66.7 | 0 | 0.0 | 0 | 0.0 | 0 | 0.0 | 0 | 0.0 | 0 | 0.0 |
|  |  | **Pain Intensity** | moderate | 1 | 11.1 | 0 | 0.0 | 0 | 0.0 | 0 | 0.0 | 0 | 0.0 | 0 | 0.0 | 0 | 0.0 |
|  |  |  | severe | 7 | 77.8 | 2 | 66.7 | 1 | 100.0 | 0 | 0.0 | 0 | 0.0 | 0 | 0.0 | 0 | 0.0 |
|  |  | **Trigger** | present | 5 | 55.6 | 0 | 0.0 | 1 | 100.0 | 0 | 0.0 | 1 | 100.0 | 0 | 0.0 | 0 | 0.0 |
|  |  | **CAS** | present | 2 | 22.2 | 0 | 0.0 | 0 | 0.0 | 0 | 0.0 | 0 | 0.0 | 0 | 0.0 | 0 | 0.0 |
|  |  | **SD** | present | 1 | 11.1 | 2 | 66.7 | 0 | 0.0 | 0 | 0.0 | 0 | 0.0 | 1 | 100.0 | 0 | 0.0 |
|  |  | **Treated during visit** | no therapy | 6 | 66.7 | 3 | 100.0 | 0 | 0.0 | 0 | 0.0 | 1 | 100.0 | 1 | 100.0 | 0 | 0.0 |
|  |  |  | monoterapy | 1 | 11.1 | 0 | 0.0 | 1 | 100.0 | 0 | 0.0 | 0 | 0.0 | 0 | 0.0 | 0 | 0.0 |
|  |  |  | polytherapy | 2 | 22.2 | 0 | 0.0 | 0 | 0.0 | 0 | 0.0 | 0 | 0.0 | 0 | 0.0 | 0 | 0.0 |
|  |  | **Therapy at discharge** | no therapy | 0 | 0.0 | 1 | 33.3 | 0 | 0.0 | 0 | 0.0 | 0 | 0.0 | 0 | 0.0 | 0 | 0.0 |
|  |  |  | monoterapy | 4 | 44.4 | 0 | 0.0 | 0 | 0.0 | 0 | 0.0 | 1 | 100.0 | 0 | 0.0 | 0 | 0.0 |
|  |  |  | polytherapy | 5 | 55.6 | 2 | 66.7 | 1 | 100.0 | 0 | 0.0 | 0 | 0.0 | 1 | 100.0 | 0 | 0.0 |

a-percentages calculated by total number of patients in a selected group; TN – trigeminal neuralgia, TNp – painful trigeminal neuropathy, pON – presumed occipital neuralgia , pPIFP – presumed persistent idiopathic facial pain, GN –glossopharyngeal neuralgia; NINp –painful nervus intermedius neuropathy; pTN+pGN – presumed concomitant trigeminal and glossopharyngeal neuralgia; COV-19 – COVID-19 period; IQR – interquartile range; CAS – cranial autonomic symptoms, SD – sensory disturbances; Sums of for certain groups are not necessarily 100. Missing data is reported in the Supplementary Data Table 1

**Supplementary Data Table 7.** Treatment in the emergency department before and after COVID-19.

| **Group** | | | **Craniofacial pain type** | | | | | | | | | | | | | | | |
| --- | --- | --- | --- | --- | --- | --- | --- | --- | --- | --- | --- | --- | --- | --- | --- | --- | --- | --- |
|  |  |  | **Total** | | **TN** | | **TNp** | | **ON** | | **pPIFP** | | **GN** | | **NINp** | | **pTN+pGN** | |
|  |  |  | **n** | **%^a^** | **n** | **%^a^** | **n** | **%^a^** | **n** | **%^a^** | **n** | **%^a^** | **n** | **%^a^** | **n** | **%^a^** | **n** | **%^a^** |
| **Cov-19** | **pre** | **Coanalgesics** | 8 | 5.7 | 7 | 6.3 | 1 | 12.5 | 0 | 0.0 | 0 | 0.0 | 0 | 0.0 | 0 | 0.0 | 0 | 0.0 |
|  |  | **Carbamazepine** | 2 | 1.4 | 2 | 1.8 | 0 | 0.0 | 0 | 0.0 | 0 | 0.0 | 0 | 0.0 | 0 | 0.0 | 0 | 0.0 |
|  |  | **Diazepam** | 4 | 2.9 | 3 | 2.7 | 1 | 12.5 | 0 | 0.0 | 0 | 0.0 | 0 | 0.0 | 0 | 0.0 | 0 | 0.0 |
|  |  | **Corticosteroids** | 6 | 4.3 | 5 | 4.5 | 0 | 0.0 | 1 | 11.1 | 0 | 0.0 | 0 | 0.0 | 0 | 0.0 | 0 | 0.0 |
|  |  | Deksametazon | 2 | 1.4 | 2 | 1.8 | 0 | 0.0 | 0 | 0.0 | 0 | 0.0 | 0 | 0.0 | 0 | 0.0 | 0 | 0.0 |
|  |  | Metilprednizolon | 4 | 2.9 | 3 | 2.7 | 0 | 0.0 | 1 | 11.1 | 0 | 0.0 | 0 | 0.0 | 0 | 0.0 | 0 | 0.0 |
|  |  | **Analgesics** | 45 | 32.1 | 34 | 30.4 | 4 | 50.0 | 4 | 44.4 | 1 | 25.0 | 0 | 0.0 | 1 | 33.3 | 1 | 100.0 |
|  |  | **Tramadol** | 12 | 8.6 | 11 | 9.8 | 0 | 0.0 | 1 | 11.1 | 0 | 0.0 | 0 | 0.0 | 0 | 0.0 | 0 | 0.0 |
|  |  | **Nonopioids** | 37 | 26.4 | 27 | 24.1 | 4 | 50.0 | 3 | 33.3 | 1 | 25.0 | 0 | 0.0 | 1 | 33.3 | 1 | 100.0 |
|  |  | Paracetamol | 2 | 1.4 | 2 | 1.8 | 0 | 0.0 | 0 | 0.0 | 0 | 0.0 | 0 | 0.0 | 0 | 0.0 | 0 | 0.0 |
|  |  | Ketoprofen | 1 | 0.7 | 1 | 0.9 | 0 | 0.0 | 0 | 0.0 | 0 | 0.0 | 0 | 0.0 | 0 | 0.0 | 0 | 0.0 |
|  |  | Diklofenak | 6 | 4.3 | 4 | 3.6 | 1 | 12.5 | 1 | 11.1 | 0 | 0.0 | 0 | 0.0 | 0 | 0.0 | 0 | 0.0 |
|  |  | Ketorolak | 22 | 15.7 | 17 | 15.2 | 2 | 25.0 | 0 | 0.0 | 1 | 25.0 | 0 | 0.0 | 1 | 33.3 | 1 | 100.0 |
|  |  | Metamizol | 10 | 7.1 | 7 | 6.3 | 1 | 12.5 | 2 | 22.2 | 0 | 0.0 | 0 | 0.0 | 0 | 0.0 | 0 | 0.0 |
|  |  | **Other** (mannitol, O2) | 13 | 9.3 | 10 | 8.9 | 1 | 12.5 | 2 | 22.2 | 0 | 0.0 | 0 | 0.0 | 0 | 0.0 | 0 | 0.0 |
|  | **post** | **Deksametazon** | 1 | 6.7 | 1 | 11.1 | 0 | 0.0 | 0 | 0.0 | 0 | 0.0 | 0 | 0.0 | 0 | 0.0 | 0 | 0.0 |
|  |  | **Analgesics** | 3 | 20.0 | 2 | 22.2 | 0 | 0.0 | 1 | 100.0 | 0 | 0.0 | 0 | 0.0 | 0 | 0.0 | 0 | 0.0 |
|  |  | **Tramadol** | 1 | 6.7 | 1 | 11.1 | 0 | 0.0 | 0 | 0.0 | 0 | 0.0 | 0 | 0.0 | 0 | 0.0 | 0 | 0.0 |
|  |  | **Nonopioid** | 4 | 26.7 | 3 | 33.3 | 0 | 0.0 | 1 | 100.0 | 0 | 0.0 | 0 | 0.0 | 0 | 0.0 | 0 | 0.0 |
|  |  | Diklofenak+nimesulid | 2 | 13.3 | 1 | 11.1 | 0 | 0.0 | 1 | 100.0 | 0 | 0.0 | 0 | 0.0 | 0 | 0.0 | 0 | 0.0 |
|  |  | Ketorolak+metamizol | 1 | 6.7 | 1 | 11.1 | 0 | 0.0 | 0 | 0.0 | 0 | 0.0 | 0 | 0.0 | 0 | 0.0 | 0 | 0.0 |
|  |  | Ketorolak | 1 | 6.7 | 1 | 11.1 | 0 | 0.0 | 0 | 0.0 | 0 | 0.0 | 0 | 0.0 | 0 | 0.0 | 0 | 0.0 |
|  |  | **Other** (mannitol, O2) | 2 | 13.3 | 2 | 22.2 | 0 | 0.0 | 0 | 0.0 | 0 | 0.0 | 0 | 0.0 | 0 | 0.0 | 0 | 0.0 |

a-percentages calculated by total number of patients in a selected group; TN – trigeminal neuralgia, TNp – painful trigeminal neuropathy, pON – presumed occipital neuralgia , pPIFP – presumed persistent idiopathic facial pain, GN –glossopharyngeal neuralgia; NINp –painful nervus intermedius neuropathy; pTN+pGN – presumed concomitant trigeminal and glossopharyngeal neuralgia; COV-19 – COVID-19 period; Sums of for certain groups are not necessarily 100. Missing data is reported in the Supplementary Data Table 1

**Supplementary Data Table 8.** Treatment at discharge before and after COVID-19 outbreak.

| **Group** | | | **Craniofacial pain type** | | | | | | | | | | | | | | | |
| --- | --- | --- | --- | --- | --- | --- | --- | --- | --- | --- | --- | --- | --- | --- | --- | --- | --- | --- |
|  |  |  | **Total** | | **TN** | | **TNp** | | **ON** | | **pPIFP** | | **GN** | | **NINp** | | **pTN+pGN** | |
|  |  |  | **n** | **%^a^** | **n** | **%^a^** | **n** | **%^a^** | **n** | **%^a^** | **n** | **%^a^** | **n** | **%^a^** | **n** | **%^a^** | **n** | **%^a^** |
| **COV-19** | **pre** | **Coanalgesics** | 123 | 87.9 | 101 | 90.2 | 8 | 100.0 | 7 | 77.8 | 2 | 50.0 | 3 | 100.0 | 1 | 33.3 | 1 | 100.0 |
|  |  | **Anticonvulsives** | 119 | 85.0 | 98 | 87.5 | 8 | 100.0 | 7 | 77.8 | 2 | 50.0 | 3 | 100.0 | 0 | 0.0 | 1 | 100.0 |
|  |  | Carbamazepine | 96 | 68.6 | 80 | 71.4 | 4 | 50.0 | 6 | 66.7 | 2 | 50.0 | 3 | 100.0 | 0 | 0.0 | 1 | 100.0 |
|  |  | Gabapentine | 15 | 10.7 | 14 | 12.5 | 1 | 12.5 | 0 | 0.0 | 0 | 0.0 | 0 | 0.0 | 0 | 0.0 | 0 | 0.0 |
|  |  | Pregabalin | 18 | 12.9 | 13 | 11.6 | 3 | 37.5 | 2 | 22.2 | 0 | 0.0 | 0 | 0.0 | 0 | 0.0 | 0 | 0.0 |
|  |  | Klonazepam | 1 | 0.7 | 0 | 0.0 | 1 | 12.5 | 0 | 0.0 | 0 | 0.0 | 0 | 0.0 | 0 | 0.0 | 0 | 0.0 |
|  |  | Levetiracetam | 1 | 0.7 | 1 | 0.9 | 0 | 0.0 | 0 | 0.0 | 0 | 0.0 | 0 | 0.0 | 0 | 0.0 | 0 | 0.0 |
|  |  | Valproate | 1 | 0.7 | 1 | 0.9 | 0 | 0.0 | 0 | 0.0 | 0 | 0.0 | 0 | 0.0 | 0 | 0.0 | 0 | 0.0 |
|  |  | **Amytriptiline** | 12 | 8.6 | 9 | 8.0 | 2 | 25.0 | 0 | 0.0 | 0 | 0.0 | 0 | 0.0 | 1 | 33.3 | 0 | 0.0 |
|  |  | **Benzodiazepines** | 10 | 7.1 | 6 | 5.4 | 0 | 0.0 | 1 | 11.1 | 1 | 25.0 | 1 | 33.3 | 1 | 33.3 | 0 | 0.0 |
|  |  | Diazepam | 4 | 2.9 | 3 | 2.7 | 0 | 0.0 | 1 | 11.1 | 0 | 0.0 | 0 | 0.0 | 0 | 0.0 | 0 | 0.0 |
|  |  | Bromazepam | 5 | 3.6 | 3 | 2.7 | 0 | 0.0 | 0 | 0.0 | 1 | 25.0 | 1 | 33.3 | 0 | 0.0 | 0 | 0.0 |
|  |  | Lorazepam | 1 | 0.7 | 0 | 0.0 | 0 | 0.0 | 0 | 0.0 | 0 | 0.0 | 0 | 0.0 | 1 | 33.3 | 0 | 0.0 |
|  |  | **Corticosteroids** | 3 | 2.1 | 3 | 2.7 | 0 | 0.0 | 0 | 0.0 | 0 | 0.0 | 0 | 0.0 | 0 | 0.0 | 0 | 0.0 |
|  |  | Dexamethasone | 2 | 1.4 | 2 | 1.8 | 0 | 0.0 | 0 | 0.0 | 0 | 0.0 | 0 | 0.0 | 0 | 0.0 | 0 | 0.0 |
|  |  | Methylprednisolone | 1 | 0.7 | 1 | 0.9 | 0 | 0.0 | 0 | 0.0 | 0 | 0.0 | 0 | 0.0 | 0 | 0.0 | 0 | 0.0 |
|  |  | **Analgesics** | 57 | 40.7 | 40 | 35.7 | 6 | 75.0 | 5 | 55.6 | 4 | 100.0 | 1 | 33.3 | 1 | 33.3 | 0 | 0.0 |
|  |  | **Opioids** | 19 | 13.6 | 16 | 14.3 | 1 | 12.5 | 2 | 22.2 | 0 | 0.0 | 0 | 0.0 | 0 | 0.0 | 0 | 0.0 |
|  |  | Tramadol | 18 | 12.9 | 15 | 13.4 | 1 | 12.5 | 2 | 22.2 | 0 | 0.0 | 0 | 0.0 | 0 | 0.0 | 0 | 0.0 |
|  |  | Fentanyl | 1 | 0.7 | 1 | 0.9 | 0 | 0.0 | 0 | 0.0 | 0 | 0.0 | 0 | 0.0 | 0 | 0.0 | 0 | 0.0 |
|  |  | **Nonopioids** | 48 | 34.3 | 32 | 28.6 | 5 | 62.5 | 5 | 55.6 | 4 | 100.0 | 1 | 33.3 | 1 | 33.3 | 0 | 0.0 |
|  |  | Paracetamol | 26 | 18.6 | 16 | 14.3 | 3 | 37.5 | 3 | 33.3 | 3 | 75.0 | 1 | 33.3 | 0 | 0.0 | 0 | 0.0 |
|  |  | Paracetamol and  Ibuprofen (single pill) | 6 | 4.3 | 5 | 4.5 | 0 | 0.0 | 1 | 11.1 | 0 | 0.0 | 0 | 0.0 | 0 | 0.0 | 0 | 0.0 |
|  |  | Ibuprofen | 9 | 6.4 | 4 | 3.6 | 0 | 0.0 | 3 | 33.3 | 2 | 50.0 | 0 | 0.0 | 0 | 0.0 | 0 | 0.0 |
|  |  | Dexketoprofen | 1 | 0.7 | 0 | 0.0 | 0 | 0.0 | 0 | 0.0 | 1 | 25.0 | 0 | 0.0 | 0 | 0.0 | 0 | 0.0 |
|  |  | Naproxen | 2 | 1.4 | 1 | 0.9 | 0 | 0.0 | 1 | 11.1 | 0 | 0.0 | 0 | 0.0 | 0 | 0.0 | 0 | 0.0 |
|  |  | Diclofenac | 2 | 1.4 | 2 | 1.8 | 0 | 0.0 | 0 | 0.0 | 0 | 0.0 | 0 | 0.0 | 0 | 0.0 | 0 | 0.0 |
|  |  | Ketorolac | 8 | 5.7 | 7 | 6.25 | 0 | 0.0 | 0 | 0.0 | 0 | 0.0 | 0 | 0.0 | 1 | 33.3 | 0 | 0.0 |
|  |  | Metamizole | 4 | 2.9 | 2 | 1.8 | 1 | 12.5 | 1 | 11.1 | 0 | 0.0 | 0 | 0.0 | 0 | 0.0 | 0 | 0.0 |
|  |  | **CAM** | 30 | 21.4 | 24 | 21.4 | 2 | 25.0 | 0 | 0.0 | 2 | 50.0 | 0 | 0.0 | 2 | 66.7 | 0 | 0.0 |
|  |  | Capsaicine | 3 | 2.1 | 2 | 1.8 | 1 | 12.5 | 0 | 0.0 | 0 | 0.0 | 0 | 0.0 | 0 | 0.0 | 0 | 0.0 |
|  | post | **Coanalgesics** | 13 | 86.7 | 9 | 100.0 | 2 | 66.7 | 1 | 100.0 | 0 | 0.0 | 1 | 100.0 | 0 | 0.0 | 0 | 0.0 |
|  |  | **Anticonvulsives** | 12 | 80.0 | 9 | 100.0 | 1 | 33.3 | 1 | 100.0 | 0 | 0.0 | 1 | 100.0 | 0 | 0.0 | 0 | 0.0 |
|  |  | Carbamazepine | 9 | 60.0 | 7 | 77.8 | 1 | 33.3 | 0 | 0.0 | 0 | 0.0 | 1 | 100.0 | 0 | 0.0 | 0 | 0.0 |
|  |  | Gabapentine | 1 | 6.7 | 1 | 11.1 | 0 | 0.0 | 0 | 0.0 | 0 | 0.0 | 0 | 0.0 | 0 | 0.0 | 0 | 0.0 |
|  |  | Pregabalin | 3 | 20.0 | 1 | 11.1 | 1 | 33.3 | 1 | 100.0 | 0 | 0.0 | 0 | 0.0 | 0 | 0.0 | 0 | 0.0 |
|  |  | **Diazepam** | 1 | 6.7 | 0 | 0.0 | 0 | 0.0 | 0 | 0.0 | 0 | 0.0 | 0 | 0.0 | 1 | 100.0 | 0 | 0.0 |
|  |  | **Dexamethasone** | 1 | 6.7 | 0 | 0.0 | 0 | 0.0 | 0 | 0.0 | 0 | 0.0 | 0 | 0.0 | 1 | 100.0 | 0 | 0.0 |
|  |  | **Analgesics** | 7 | 46.7 | 5 | 55.6 | 1 | 33.3 | 0 | 0.0 | 0 | 0.0 | 0 | 0.0 | 1 | 100.0 | 0 | 0.0 |
|  |  | **Tramadol** | 2 | 13.3 | 2 | 22.2 | 0 | 0.0 | 0 | 0.0 | 0 | 0.0 | 0 | 0.0 | 0 | 0.0 | 0 | 0.0 |
|  |  | **Neopioids** | 8 | 53.3 | 6 | 66.7 | 1 | 33.3 | 0 | 0.0 | 0 | 0.0 | 0 | 0.0 | 1 | 100.0 | 0 | 0.0 |
|  |  | Paracetamol | 5 | 33.3 | 5 | 55.6 | 0 | 0.0 | 0 | 0.0 | 0 | 0.0 | 0 | 0.0 | 0 | 0.0 | 0 | 0.0 |
|  |  | Paracetamol and  Ibuprofen (single pill) | 1 | 6.7 | 0 | 0.0 | 1 | 33.3 | 0 | 0.0 | 0 | 0.0 | 0 | 0.0 | 0 | 0.0 | 0 | 0.0 |
|  |  | Ketorolac | 1 | 6.7 | 1 | 11.1 | 0 | 0.0 | 0 | 0.0 | 0 | 0.0 | 0 | 0.0 | 0 | 0.0 | 0 | 0.0 |
|  |  | **CAM** | 7 | 46.7 | 4 | 44.4 | 1 | 33.3 | 1 | 100.0 | 0 | 0.0 | 0 | 0.0 | 1 | 100.0 | 0 | 0.0 |

a-percentages calculated by total number of patients in a selected group; TN – trigeminal neuralgia, TNp – painful trigeminal neuropathy, pON – presumed occipital neuralgia , pPIFP – presumed persistent idiopathic facial pain, GN –glossopharyngeal neuralgia; NINp –painful nervus intermedius neuropathy; pTN+pGN – presumed concomitant trigeminal and glossopharyngeal neuralgia; COV-19 – COVID-19 period; Sums of for certain groups are not necessarily 100. Missing data is reported in the Supplementary Data Table 1

**Supplementary Data Table 9.** Clinical characteristics of selected craniofacial pain categories before and after COVID-19.

| **Group** | | **COV-19** | | | | | | | | | | | |
| --- | --- | --- | --- | --- | --- | --- | --- | --- | --- | --- | --- | --- | --- |
|  |  | **pre** | | | | | | **post** | | | | | |
|  |  | **TN** | | **TNp** | | **pPIFP** | | **TN** | | **TNp** | | **pPIFP** | |
|  |  | **n** | **%^a^** | **n** | **%^a^** | **n** | **%^a^** | **n** | **%^a^** | **n** | **%^a^** | **n** | **%^a^** |
| **Distribution^b^** | V1 | 9 | 8.0 | 4 | 50.0 | 0 | 0.0 | 1 | 11.1 | 3 | 100.0 | 0 | 0.0 |
|  | V2 | 30 | 26.8 | 3 | 37.5 | 0 | 0.0 | 0 | 0.0 | 0 | 0.0 | 0 | 0.0 |
|  | V3 | 17 | 15.2 | 0 | 0.0 | 1 | 25.0 | 3 | 33.3 | 0 | 0.0 | 0 | 0.0 |
|  | V1+V2 | 12 | 10.7 | 1 | 12.5 | 0 | 0.0 | 0 | 0.0 | 0 | 0.0 | 0 | 0.0 |
|  | V2+V3 | 28 | 25.0 | 0 | 0.0 | 1 | 25.0 | 4 | 44.4 | 0 | 0.0 | 0 | 0.0 |
|  | V1+V3 | 0 | 0.0 | 0 | 0.0 | 0 | 0.0 | 1 | 11.1 | 0 | 0.0 | 0 | 0.0 |
|  | V1-V3 | 8 | 7.1 | 0 | 0.0 | 1 | 25.0 | 0 | 0.0 | 0 | 0.0 | 0 | 0.0 |
|  | extratrigeminal | 3 | 2.7 | 0 | 0.0 | 1 | 25.0 | 0 | 0.0 | 0 | 0.0 | 0 | 0.0 |
| **Triggers** | speech | 26 | 23.2 | 1 | 12.5 | 0 | 0.0 | 1 | 11.1 | 0 | 0.0 | 0 | 0.0 |
|  | facial expressions | 9 | 8.0 | 2 | 25.0 | 0 | 0.0 | 1 | 11.1 | 0 | 0.0 | 0 | 0.0 |
|  | chewing | 39 | 34.8 | 2 | 25.0 | 0 | 0.0 | 2 | 22.2 | 0 | 0.0 | 0 | 0.0 |
|  | cold | 8 | 7.1 | 0 | 0.0 | 0 | 0.0 | 1 | 11.1 | 0 | 0.0 | 0 | 0.0 |
|  | touch | 27 | 24.1 | 0 | 0.0 | 0 | 0.0 | 0 | 0.0 | 0 | 0.0 | 0 | 0.0 |
|  | weather condition change | 11 | 9.8 | 1 | 12.5 | 2 | 50.0 | 2 | 22.2 | 0 | 0.0 | 0 | 0.0 |
|  | tooth brushing | 8 | 7.1 | 1 | 12.5 | 0 | 0.0 | 1 | 11.1 | 0 | 0.0 | 0 | 0.0 |
|  | jaw opening | 18 | 16.1 | 0 | 0.0 | 0 | 0.0 | 1 | 11.1 | 0 | 0.0 | 0 | 0.0 |
|  | face washing | 8 | 7.1 | 0 | 0.0 | 0 | 0.0 | 0 | 0.0 | 0 | 0.0 | 0 | 0.0 |
|  | hot beverages | 2 | 1.8 | 0 | 0.0 | 0 | 0.0 | 1 | 11.1 | 0 | 0.0 | 0 | 0.0 |
|  | swallowing | 9 | 8.0 | 0 | 0.0 | 0 | 0.0 | 1 | 11.1 | 0 | 0.0 | 0 | 0.0 |
|  | minimal strains and movements | 4 | 3.6 | 0 | 0.0 | 1 | 25.0 | 0 | 0.0 | 0 | 0.0 | 0 | 0.0 |
|  | coughing | 1 | 0.9 | 0 | 0.0 | 0 | 0.0 | 0 | 0.0 | 0 | 0.0 | 0 | 0.0 |
| **Pain description** | stabbing | 25 | 22.3 | 1 | 12.5 | 0 | 0.0 | 3 | 33.3 | 0 | 0.0 | 0 | 0.0 |
|  | dull | 2 | 1.8 | 1 | 12.5 | 2 | 50.0 | 0 | 0.0 | 1 | 33.3 | 0 | 0.0 |
|  | shock-like | 26 | 23.2 | 1 | 12.5 | 0 | 0.0 | 1 | 11.1 | 0 | 0.0 | 0 | 0.0 |
|  | sharp | 7 | 6.3 | 0 | 0.0 | 0 | 0.0 | 0 | 0.0 | 0 | 0.0 | 0 | 0.0 |
|  | burning | 2 | 1.8 | 2 | 25.0 | 0 | 0.0 | 0 | 0.0 | 1 | 33.3 | 0 | 0.0 |
|  | throbbing | 5 | 4.5 | 0 | 0.0 | 0 | 0.0 | 1 | 11.1 | 1 | 33.3 | 0 | 0.0 |
|  | neuralgic | 10 | 8.9 | 0 | 0.0 | 0 | 0.0 | 1 | 11.1 | 0 | 0.0 | 0 | 0.0 |
|  | atypical | 2 | 1.8 | 0 | 0.0 | 1 | 25.0 | 0 | 0.0 | 0 | 0.0 | 0 | 0.0 |
|  | neuropathic | 0 | 0.0 | 1 | 12.5 | 0 | 0.0 | 0 | 0.0 | 0 | 0.0 | 0 | 0.0 |
| **CAS** | nausea and vomiting | 2 | 1.8 | 1 | 12.5 | 0 | 0.0 | 0 | 0.0 | 0 | 0.0 | 0 | 0.0 |
|  | facial flushing | 1 | 0.9 | 0 | 0.0 | 0 | 0.0 | 0 | 0.0 | 0 | 0.0 | 0 | 0.0 |
|  | facial swelling | 1 | 0.9 | 0 | 0.0 | 0 | 0.0 | 0 | 0.0 | 0 | 0.0 | 0 | 0.0 |
|  | salivation | 1 | 0.9 | 0 | 0.0 | 0 | 0.0 | 0 | 0.0 | 0 | 0.0 | 0 | 0.0 |
|  | hypertension | 1 | 0.9 | 0 | 0.0 | 0 | 0.0 | 0 | 0.0 | 0 | 0.0 | 0 | 0.0 |
|  | lacrimation | 10 | 8.9 | 1 | 12.5 | 0 | 0.0 | 0 | 0.0 | 0 | 0.0 | 0 | 0.0 |
|  | photophobia | 1 | 0.9 | 0 | 0.0 | 0 | 0.0 | 0 | 0.0 | 0 | 0.0 | 0 | 0.0 |
|  | conjunctival hyperaemia | 1 | 0.9 | 0 | 0.0 | 0 | 0.0 | 0 | 0.0 | 0 | 0.0 | 0 | 0.0 |
|  | rhinorrhea | 3 | 2.7 | 1 | 12.5 | 0 | 0.0 | 1 | 11.1 | 0 | 0.0 | 0 | 0.0 |
| **SD** | allodynia | 10 | 8.9 | 0 | 0.0 | 0 | 0.0 | 0 | 0.0 | 0 | 0.0 | 0 | 0.0 |
|  | hyperesthesia | 12 | 10.7 | 1 | 12.5 | 0 | 0.0 | 0 | 0.0 | 0 | 0.0 | 0 | 0.0 |
|  | hypoaesthesia | 6 | 5.4 | 2 | 25.0 | 0 | 0.0 | 0 | 0.0 | 0 | 0.0 | 0 | 0.0 |
|  | dysesthesia | 9 | 8.0 | 1 | 12.5 | 1 | 25.0 | 0 | 0.0 | 1 | 33.3 | 0 | 0.0 |
|  | paresthesia | 8 | 7.1 | 0 | 0.0 | 1 | 25.0 | 1 | 11.1 | 1 | 33.3 | 0 | 0.0 |

a-percentages calculated by total number of patients in a category. b-approximated to trigeminal divisions where possible. COV-19 – COVID-19 periods;TN – trigeminal neuralgia, TNp –painful trigeminal neuropathy, pPIFP – presumed persistent idiopathic facial pain, V1-3 – trigeminal nerve divisions; CAS – Cranial autonomic symptoms, SD – sensory disturbances; Sums of % for certain groups are not necessarily 100. Missing data is reported in the Supplementary Data Table 1.

**Supplementary Data Table 10.** Difference in the number of patients before and after COVID-19

| All patients | N | Observed Proportion | Test Proportion | Exact Sig. (1-tailed)^*^ |
| --- | --- | --- | --- | --- |
| pre COVID-19 | 126 | 0.81 | 0.69 | 0.001 |
| post COVID-19 | 30 | 0.19 | 0.31 |  |
| Total | 156 | 1.00 |  |  |
| Trigeminal neuralgia | N | Observed Proportion | Test Proportion | Exact Sig. (1-tailed)^*^ |
| pre COVID-19 | 100 | 0.82 | 0.69 | 0.001 |
| post COVID-19 | 22 | 0.18 | 0.31 |  |
| Total | 122 | 1.00 |  |  |

*Binomial test

### Etiology and distribution of different CFP

There were 27 TN patients (24.1%) in the pre COVID-19 group with identified etiology. They were divided into classical (8.9%), secondary (6.3%) and idiopathic (8.9%) TN. There were 2 patients (22.2%) with TN in the post COVID-19 group with identified etiology: one with classical (11.1%) and one with secondary (11.1%) TN. The etiology of the only secondary TN in the post COVID-19 group was postinflammatory trigeminal nerve enlargement. The majority of the patients remained undifferentiated TN, both in the pre (75.9%) and post COVID19 group (77.8%). None of the patients in the post COVID-19 TN group had history of invasive treatment.

All patients with TNp in the post COVID-19 group were males. Two of them had TNp attributed to the Varicella zoster virus (VZV) and one had TNp caused by a space occupying lesion. V1 branch was affected in all cases.

One female patient had right-sided pON in the distribution of the major occipital nerve. One male patient had right sided GN with oropharyngeal pain. One female patient had left sided Ramsay Hunt syndrome.
